# Detected Shifts Towards Drought‐Adaptive Strategies in the Amazon Forest Over the Last Four Decades

**DOI:** 10.1111/gcb.70727

**Published:** 2026-02-04

**Authors:** Milton Barbosa, Renata A. Maia, Imma Oliveras Menor, Ben Hur Marimon Junior, Beatriz Schwantes Marimon, G. Wilson Fernandes, Yadvinder Malhi, Jesús Aguirre‐Gutiérrez

**Affiliations:** ^1^ Biodiversity and Earth Observation, Environmental Change Institute, School of Geography and the Environment University of Oxford Oxford UK; ^2^ Department of Genetics, Ecology & Evolution Federal University of Minas Gerais Belo Horizonte Brazil; ^3^ AMAP (Botanique et Modélisation de l'Architecture des Plantes et des Végétations) Université de Montpellier, CIRAD, CNRS, INRAE IRD Montpellier France; ^4^ Environmental Change Institute, School of Geography and the Environment University of Oxford Oxford UK; ^5^ Department of Biological Sciences State University of Mato Grosso (UNEMAT) Nova Xavantina Brazil; ^6^ Knowledge Center for Biodiversity Belo Horizonte Brazil; ^7^ Leverhulme Centre for Nature Recovery University of Oxford Oxford UK

**Keywords:** climate stress, functional traits, Landsat time series, reflectance stability, remote sensing, sclerophylly, specific leaf area, spectral variability

## Abstract

The Amazon Forest is undergoing rapid ecological shifts driven by intensifying drought, rising temperatures, and widespread anthropogenic disturbance. Yet the reorganization of vegetation functional strategies under climate stress remains poorly quantified at the biome scale. Here, we show that the temporal stability of canopy reflectance offers a sensitive remote proxy for sclerophylly—leaf toughness, a key indicator of conservative, drought‐adaptive plant strategies. By integrating ground‐based trait data (specific leaf area, SLA) from over 3000 trees across 448 plots in the Amazon‐Cerrado savanna transition zone with high‐resolution remote sensing imagery, we demonstrate that lower SLA—a well‐established proxy for conservative leaf strategies—is associated with reduced dry‐season variability in the blue band spectral reflectance of vegetation. Extending the analysis across 130 plots in nine Amazonian countries using 40 years of harmonised remote sensing data, we find that dry‐season reflectance variability has declined by ~34% (a drop of ~10 percentage points) since 1984, indicating a biome‐wide shift toward greater drought tolerance. This trend is most pronounced in the southern and eastern Amazon and closely tracks rising climate stress, particularly increased temperature, evaporative demand, and water deficit. If these patterns persist, much of the southern and eastern Amazon could reach reflectance‐stability levels comparable to transitional zones with the Cerrado savanna biome within the next three to four decades. Our results show signals of an early‐stage forest functional transformation that could reduce forest productivity and carbon uptake, increase vulnerability to fire, and diminish biodiversity. These findings highlight regions where early signs of reduced forest resilience are emerging, underscoring the need for spatially targeted conservation.

## Graphical abstract

Amazon forests are facing hotter, drier conditions, and we used 40 years of satellite data to look for early signs of ecological change. Combining satellite observations with leaf measurements from more than 3000 trees, we show that many forests now show smaller dry‐season fluctuations in canopy colour—especially in the southern and eastern Amazon. This growing dry‐season stability is consistent with a shift toward tougher, more drought‐tolerant vegetation, with potential consequences for carbon storage and increasing vulnerability to fire.

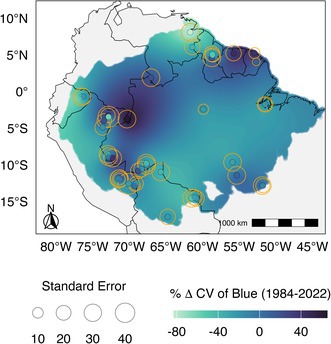

## 1 Introduction

The Amazon forest, a cornerstone of global biodiversity and climate regulation, faces escalating threats from deforestation, anthropogenic burning, and progressive climate change (Brienen et al. 2015; Cochrane and Laurance 2008; Cox et al. 2004; Flores et al. 2024; Malhi et al. 2009). These pressures are pushing the biome towards a critical tipping point, where dense, moist forests gradually transition into open, fire‐prone landscapes losing their original biodiversity and functional integrity (Veldman 2016; Veldman and Putz 2011). This transition involves structural and functional shifts in vegetation, particularly the increased dominance of drought‐tolerant species, with implications for global carbon cycling and regional climate feedbacks (Espinoza et al. 2024; Griscom et al. 2017). While evidence of such changes is growing, the mechanisms and extent to which they are unfolding across the Amazon remain poorly understood, especially in relation to spatial and temporal variation in climatic and anthropogenic stressors (Aguirre‐Gutiérrez et al. 2022; Aleixo et al. 2019; Staal et al. 2018). This complex process may involve intermediate stages, such as the transition from rainforests to deciduous forests, eventually leading to the complete loss of forest cover over the long term (Esquivel‐Muelbert et al. 2019; Flores et al. 2024; Staal et al. 2018). As conditions become warmer and drier, species better adapted to water‐limited environments may become more prevalent (Aguirre‐Gutiérrez et al. 2019; Ahrens et al. 2019). These species often share a suite of traits associated with conservative ecological strategies, including low specific leaf area (SLA)—the ratio of leaf area to dry mass, robust hydraulic safety margins and characteristic leaf phenologies that limit exposure to peak water stress, as documented for the Cerrado savanna biome and other seasonally dry tropical systems (Alberton et al. 2019; Cianciaruso et al. 2013; de Camargo et al. 2018; Franco 2002; Reich 2014; Vico et al. 2015; Wright et al. 2004).

Sclerophylly, broadly defined, is not a single trait but an emergent plant strategy characterised by thick, tough leaves, long lifespans, slow growth rates, and synchronised phenological events that enhance survival under chronic stress (Chabot and Hicks 1982; Reich 2014; Westoby et al. 2002; Wright et al. 2004; Wright and Cannon 2001). This strategy is typically observed in vegetation adapted to seasonal drought, nutrient‐poor soils, and frequent disturbance, such as savannas and Mediterranean (Cianciaruso et al. 2013; de Camargo et al. 2018; Rossatto et al. 2013). In the Amazon, the rise of such strategy may signal adaptive shifts in response to intensified climate stress (Aleixo et al. 2019; Flores et al. 2024; Lovejoy and Nobre 2019). Among the suite of traits that define sclerophyllous strategies, SLA is particularly informative. SLA sits at the core of the global Leaf Economics Spectrum, covarying with other structural, physiological, and phenological features that define plant resource‐use strategies (Díaz et al. 2016; Poorter et al. 2009; Reich 2014; Wright et al. 2004). While SLA is not equivalent to sclerophylly, it serves as a robust, field‐measurable proxy for the broader acquisitive‐conservative continuum (Poorter et al. 2009). We therefore use SLA as an anchor trait for interpreting variation in drought‐adaptive strategies.

As the system shifts along this drought‐adaptation continuum, changes in leaf traits and phenology—from evergreen leaf exchange towards more seasonal leaf loss—may emerge as early, detectable signatures of functional reorganization. In seasonally dry ecosystems, leaf habit and phenology generate distinct windows of canopy change versus stability. Evergreen sclerophyllous species retain tough, long‐lived leaves through the dry season and typically exhibit low leaf turnover, whereas fully deciduous phenologies—more typical of strongly seasonal forests and drier transitions—remain leafless for much of the dry season, such that the largest changes are concentrated in the leaf‐off and leaf‐on transitions, while conditions between these events can be comparatively stable until refoliation (Chabot and Hicks 1982; Eamus 1999; Franco 2002). Semi‐ and brevideciduous species adopt intermediate strategies, with partial or brief canopy loss and leaf flushing concentrated around the transition from dry to wet conditions (Eamus 1999; Franco 2002). These phenological schedules determine when—and for how long—the canopy undergoes rapid structural and physiological change versus relative stability and, together with coordinated variation in SLA and leaf toughness, reflect broader “fast‐slow” whole‐plant strategies for water use and carbon gain in seasonally dry systems (Chabot and Hicks 1982; Reich 2014; Rossatto et al. 2013; Vico et al. 2015; Westoby et al. 2002; Wright and Cannon 2001). Because leaf optical properties respond to pigments, water status, structural carbohydrates, and surface morphology (e.g., cuticle thickness, air spaces, epicuticular waxes) and scale from leaf to canopy, this coordination should leave a detectable imprint on canopy reflectance dynamics (Asner and Martin 2008; Castro‐Esau et al. 2006; Jacquemoud and Ustin 2019; Knapp and Carter 1998).

Remote sensing studies have linked sclerophylly to distinct spectral patterns, especially in the near‐infrared (NIR) and shortwave bands (Castro‐Esau et al. 2006; Jacquemoud and Ustin 2019). However, most studies focus on mean reflectance levels, while short‐term (within‐season) stability in canopy reflectance—a potential emergent property of conservative strategies—remains underexplored (Boulton et al. 2022). We therefore hypothesise that conservative, long‐lived and phenologically buffered canopies exhibit lower within‐season (dry‐season) variability in spectral signatures than fast‐cycling canopies. This reduced short‐term variability may arise either because evergreen sclerophyllous species maintain a relatively stable leaf cohort during the dry season, or because semi‐ and brevideciduous species concentrate leaf fall and refoliation into brief transition windows, leaving a comparatively stable canopy state for most of the dry season. In both cases, changes in leaf display, pigment pools, and leaf water status are expected to be temporally clustered rather than continuous, reducing within‐season variation in canopy reflectance (Díaz et al. 2016; Knapp and Carter 1998; Reich 2014; Westoby et al. 2002; Wright and Cannon 2001). While fully deciduous phenologies occur in some Amazonian habitats, they are likely less common in the core ever‐wet evergreen Amazon.

Here, we use satellite reflectance time series and field trait data to test whether temporal stability in canopy reflectance can serve as a remote signature of sclerophylly, reflecting the dominance of conservative vegetation strategies. We specifically examine the coefficient of variation (CV) of blue reflectance, which we find to be most closely associated with variation in SLA across seasons. Our study addresses three main questions:

- Does increased sclerophylly—measured via SLA—lead to greater temporal stability in canopy reflectance?
- Has reflectance stability increased across the Amazon over the past four decades, indicating a biome‐wide shift toward more conservative strategies?
- Are these trends spatially associated with changes in climate, such as increased drought intensity and evaporative demand?

By focusing on reflectance stability, rather than absolute values, and interpreting spectral dynamics through the lens of whole‐plant strategies, we offer a new framework for tracking vegetation adaptation to climate change in tropical forests.

## 2 Materials and Methods

### 2.1 Study Area and Vegetation Plot Data

We used a two‐tiered approach combining high‐resolution, pixel‐matched analysis at the Amazon‐Cerrado transition site of Nova Xavantina with a coarser‐resolution, Amazon‐wide analysis to test how consistently vegetation strategies are expressed in canopy spectral signatures across scales. Vegetation trait data were obtained from 134 permanent forest plots, including four intensive study plots in Nova Xavantina (Mato Grosso, Brazil; −52.352° W, −14.708° S) and 130 plots distributed across 44 regions within the Amazon basin. These regions are clusters of geographically proximate plots belonging to the same landscape and were included as a higher‐level spatial unit in the hierarchical model to capture broad‐scale spatial structure and shared bioclimatic context among plots. They span nine countries—Brazil, Bolivia, Colombia, Ecuador, French Guiana, Guyana, Peru, Suriname, and Venezuela—and were selected based on data available from Sullivan et al. (2020) (Figure S1a, Table S1). All sites were located at elevations below 400 m above sea level and were chosen to minimise direct anthropogenic disturbance, allowing for an accurate assessment of natural vegetation responses to climate variation.

The four Nova Xavantina plots lie in a transitional ecotone between the Cerrado savanna and Amazon biomes (Figure S1a) and encompass a variety of vegetation types, including rocky cerrado, typical cerrado, woody cerrado, and semi‐deciduous forest. These plots were intensively sampled, yielding functional trait data for over 3300 individual woody plants with a diameter at breast height (DBH) of 10 cm or more (Aguirre‐Gutiérrez et al. 2022). Tree inventories were used to stratify species according to their contribution to stand basal area, a proxy for crown dominance. For the most abundant species (60%–80% of basal area), three to five replicate individuals were sampled. On each selected tree, both sun and shade branches were collected, and three to five leaves per branch were measured for traits. Only sun‐exposed branches were retained for the present analysis, as they are more likely to interact directly with incoming solar radiation and therefore better reflect the spectral signal detected by satellite sensors. In more open formations such as Cerrado physiognomies, within‐crown light gradients are weaker, but sunlit foliage still contributes disproportionately to top‐of‐canopy reflectance, so we use sun‐leaf traits as our primary optical proxy. The vegetation census was carried out in 2015, with trait measurements taken from March to May 2014. Further details of measurements are given in Oliveras et al. (2020) and Gvozdevaite et al. (2018).

To integrate trait data with satellite reflectance at Nova Xavantina, each permanent plot was subdivided into a grid of 10 × 10 m subplots, matching the native spatial resolution of Sentinel‐2 imagery. Plot boundaries were established using differential GPS (DGPS) to obtain sub‐meter accuracy for all corner coordinates, and the subplot grid was constructed directly from these surveyed points, ensuring consistent orientation and correspondence with the Sentinel‐2 pixel geometry (Figure S1b,c). Individual stems and crowns were geolocated within the same high‐precision reference frame: crown positions and dimensions were estimated using regionally calibrated allometric equations, with crown radii anchored to DGPS‐referenced stem coordinates. Because subplot boundaries, stem locations and crown projections were all tied to a common, high‐precision geodetic base, each 10 × 10 m subplot could be matched directly to the Sentinel‐2 pixel in which it is centred, without requiring spatial resampling or corrective alignment. While Sentinel‐2 Level‐2A pixels nominally represent 10 × 10 m grid cells, the measured signal integrates reflectance over an effective footprint defined by the sensor point‐spread function, and geolocation accuracy is on the order of a few metres. Our subplot‐pixel linkage should therefore be interpreted as an approximate, rather than perfectly sharp, correspondence, but any residual mismatch is small relative to the pixel size and unlikely to affect the sign or overall strength of the trait‐reflectance relationships reported here.

Following spatial alignment, the community‐weighted mean (CWM) of specific leaf area (SLA; cm^2^/g) was calculated for each 10 × 10 m subplot. SLA values were weighted by the horizontal area of each tree crown, following the mass ratio hypothesis, which posits that ecosystem function is disproportionately influenced by the traits of dominant species (Grime 1998). This ensured that the reflectance data for each pixel reflected the integrated signal of functionally dominant canopy species. A detailed visual workflow for trait‐to‐pixel assignment is provided in Aguirre‐Gutiérrez et al. (2021). The resulting dataset for Nova Xavantina included 448 matched SLA‐reflectance observations at 10 m resolution.

For the broader Amazon‐scale analysis, we used previously published CWM‐SLA data for 130 forest plots, originally compiled by Aguirre‐Gutiérrez et al. (2025). These trait values were derived from long‐term forest inventories and functional trait collections across the RAINFOR and ForestPlots.net networks, and were calculated at a spatial resolution of 100 × 100 m using species‐level SLA values weighted by basal area. The Amazon‐wide dataset comprises closed‐canopy forest plots distributed across the Amazon biome and does not include Cerrado plots, which are represented only at the Nova Xavantina analysis site. This network spans a wide range of climatic, edaphic, and geographic gradients and provides a robust baseline for evaluating large‐scale patterns in functional composition and their relationship to environmental drivers.

### 2.2 Satellite Reflectance Data

We used two complementary satellite datasets—Sentinel‐2 and Landsat—to assess spectral stability across spatial and temporal scales. All imagery was atmospherically corrected, cloud‐screened, and harmonized to minimise sensor‐related artefacts, ensuring robust extraction of spectral signals relevant to plant functional strategies.

#### 2.2.1 High‐Resolution Analysis (Nova Xavantina)

To investigate the relationship between sclerophylly and spectral stability at high spatial (10 m) and temporal (5‐day revisit) resolution, we used Sentinel‐2 Level‐2A surface reflectance imagery (COPERNICUS/S2_SR) for the year 2019. Time series were extracted for all 448 subplots (10 × 10 m) in Nova Xavantina using the GEE JavaScript API. A strict cloud probability threshold (< 5%) was applied, and pixels affected by saturation, clouds, or shadows were masked. Subplot centroids were buffered by 5 m to generate 10 × 10 m polygons. These adjacent plots encompass a broad gradient of vegetation strategies, allowing near one‐to‐one correspondence between satellite pixels and mapped tree crowns. The analysis period (2019‐01‐01 to 2019‐12‐31) was selected as the closest available window on GEE to the field sampling dates for SLA. We used Sentinel‐2 bands at their native resolutions (10 m: blue, green, red, NIR; 20 m: red‐edge 1–4, SWIR1, SWIR2). The 20 m bands were upsampled to a 10 m grid only to allow co‐registration with the 10 m bands, but analyses were conducted at the subplot or plot level, so the effective spatial resolution is constrained by the original 20 m bands and we do not treat upsampled pixels as independent observations. In addition to the raw spectral bands, we calculated four vegetation indices—NDVI, EVI, MSAVI, and NDRE—due to their established links with canopy structure, pigment concentrations, and nutrient status (Aguirre‐Gutiérrez et al. 2021). For each band and index, we calculated the coefficient of variation (CV%) within each month as a proxy for short‐term spectral stability, defined as the standard deviation divided by the mean of all valid, cloud‐free observations for a given site‐month combination, multiplied by 100. Monthly CVs were only computed for site‐month combinations with at least two valid observations after masking; poorly sampled combinations were excluded from the analysis. We verified that the main SLA‐CV relationship was robust to data gaps and alternative sampling thresholds; full details of the cloud masking, missing‐data patterns and sensitivity analyses are provided in Appendix S1 and in Table S2.

#### 2.2.2 Biome‐Wide Analysis (Amazon Basin)

To examine the relationship between sclerophylly and spectral stability at the regional scale, we applied the same procedure described above to the 130 Amazonian forest plots (100 × 100 m) used in the broader biome‐level analysis. These plots were not individually matched to single Sentinel‐2 pixels; instead, mean reflectance values were extracted across all pixels intersecting each plot using the reduceRegions function in GEE. Sentinel‐2 Level‐2A surface reflectance data from 2019 were used for consistency with the Nova Xavantina analysis. The Nova Xavantina and Amazon‐wide Sentinel‐2 analyses represent independent tests of the relationship between SLA and reflectance variability at 10 × 10 m and 100 × 100 m spatial grains, respectively; we do not transfer a 10 m calibration to the regional analysis.

To assess decadal‐scale changes in reflectance stability across the 130 forest sites distributed throughout the Amazon biome, we used data from the harmonised Landsat Collection 2 Tier‐1 Level‐2 product with a spatial resolution of 30 m sourced via GEE. This includes calibrated, atmospherically corrected surface reflectance from Landsat 5 (TM), Landsat 7 (ETM+), and Landsat 8 (OLI), spanning from 1984 to 2022. The collection incorporates enhanced geometric and radiometric calibration, ensuring interoperability across sensors and enabling consistent, sensor‐independent analysis over time (www.usgs.gov/landsat‐missions/landsat‐collection‐2). Inter‐sensor harmonisation was validated by comparing band reflectance across overlapping operational periods for Landsat 5, 7, and 8. We extracted reflectance values from the six primary Landsat bands (blue, green, red, NIR, SWIR1, and SWIR2) within a 50 m buffer around each plot location. Reflectance was aggregated by month and season, and only cloud‐free observations were retained (cloud probability = 0%). A strict cloud, shadow, and saturation masking protocol was implemented using the QA_PIXEL and QA_RADSAT flags. Due to the lower revisit frequency of Landsat, we calculated the coefficient of variation within seasons (wet and dry) for each year, focusing particularly on the blue reflectance band. The Landsat analysis is used solely to characterise decadal trends in blue‐band CV around the forest plots; we do not directly apply the Sentinel‐2‐based SLA‐CV relationship to the Landsat time series.

### 2.3 Hydrological and Climatic Variables

To evaluate climatic correlates of functional change in the Amazon, we used monthly historical climate data from TerraClimate (Abatzoglou et al. 2018) accessed via Google Earth Engine (GEE), covering the period from 1984 to 2022. Variables included maximum and minimum temperature (tmmx, tmmn), vapour pressure deficit (vpd), wind speed (vs), downward surface shortwave radiation (srad), accumulated precipitation (pr), climate water deficit (def), soil moisture (soil), runoff (ro), evapotranspiration (aet), and palmer drought severity index (PDSI). Each of these variables plays a role in shaping the environmental conditions that can influence vegetation (see Table S5 for a brief explanation of each variable and its potential relevance to your study). TerraClimate offers high‐resolution (∼4 km) gridded data for global land areas derived from the WorldClim dataset, along with variable spatial resolution data from CRU Ts4.0 and the Japanese 55‐year Reanalysis (JRA55).

For each climatic variable (e.g., VPD, AET, DEF, PDSI, runoff), we then derived monthly climatologies as well as annual and wet/dry‐season means for each plot by averaging values over 1984–2022. These metrics were used as long‐term descriptors of local climate and hydrological stress rather than short‐term variability indices. For each plot, we defined wet and dry seasons using long‐term TerraClimate precipitation. We first computed the mean precipitation for each calendar month over 1984–2022, then ranked the 12 months by this long‐term mean. The four driest months were classified as that plot's dry season and the remaining 8 months as its wet season, allowing season boundaries to follow local rainfall regimes rather than a fixed calendar split across the basin.

Hydrological variables for the 130 Amazon sites were further characterised using data from the ECOSTRESS (Ecosystem Spaceborne Thermal Radiometer Experiment on Space Station) satellite (Fisher et al. 2020). We used the ECOSTRESS Level‐3 ET PT‐JPL product (ECO3ETPTJPL v001), extracting the evaporative stress index (ESI), water use efficiency (WUE), and instantaneous evapotranspiration (Evap). ECOSTRESS provides data with a resolution of 70 m every 1 to 5 days. This information is vital for assessing plant water stress. Data from ECOSTRESS were collected using the online AppEEARS tool (available at https://lpdaacsvc.cr.usgs.gov/appeears), covering the period from January 2019 to December 2022. This timeframe allows enough data for calculating monthly means and aligns with the collection of the functional vegetation data in our study sites in the Amazon biome.

All processed remote sensing and climate time series, together with the full Google Earth Engine workflows, are archived in Zenodo (Barbosa 2026; Barbosa et al. 2026).

### 2.4 Statistical Analysis

All statistical analyses and model fittings were conducted using the R programming language, version 4.3.0 (R Core Team 2023), and specific libraries mentioned below.

#### 2.4.1 Variable Selection

To examine relationships among predictors and guide variable preselection for model fitting, we computed Pearson correlations among all candidate variables and conducted exploratory PCAs (Figures S2, S5, S6, S10 and S11). We then assessed associations between SLA and the coefficients of variation (CV) for 14 spectral bands/indices (blue, green, red, red_edge1 to 4, nir, swir1 and 2, ndvi, evi, msavi, ndre). To avoid selection bias from choosing the strongest association across multiple bands, we (i) report Holm‐adjusted *p*‐values across the 14 tests; and (ii) performed a selection‐aware max‐|r| permutation test, which repeats the “select the maximum |r| across 14 bands” step on each permuted dataset (permuting SLA relative to the full multiband CV set, thereby preserving inter‐band dependence). We also report Fisher‐z 95% confidence intervals and a robustness check using Spearman's *ρ*.

#### 2.4.2 Spatial Autocorrelation Analysis

For all response variables in the study we tested for spatial autocorrelation. The spatial data were processed to create a spatial features object, assigning the geographical coordinate system (WGS 84) to ensure accurate spatial representation. The distance matrix, calculated in metres, was derived from the spatial features object using the *st_distance* function. The maximum distance within the study area was determined. To assess spatial autocorrelation, a spatial weight matrix (W) was constructed using the *dnearneigh* function from the *spdep* package. The distance parameter for creating the spatial weights matrix was set to the maximum distance obtained from the distance matrix. The spatial weights matrix was then converted to a *listw* object using *nb2listw*. The Moran's *I* statistic was computed through the *moran.test* function to quantify the degree of spatial autocorrelation for the response variables using the constructed spatial weights matrix. When significant spatial autocorrelation was detected, we applied various correlation structures to the model to identify the most appropriate correlation structure, using the *nlme* and *spdep* libraries. The correlation structures included exponential (*corExp*), Gaussian (*corGaus*), spherical (*corSpher*), rational quadratic (*corRatio*), and linear (*corLin*). We employed Akaike Information Criterion (AIC) to compare the goodness of fit among the different models. The model with the lowest AIC value was selected as the model with the best correlation structure.

#### 2.4.3 High‐Resolution SLA‐Reflectance Models (Nova Xavantina)

To investigate the interactive effects of SLA and seasonal variation on the reflectance of vegetation, we modelled the Sentinel‐2 derived intra‐month variation (CV) in the reflectance of each of the preselected bands and indices (blue, green, red edge 4, NIR, and NDRE) as a function of the CWM of SLA (cm^2^/g) at the pixel‐level (10 × 10 m), season (dry and wet), and their interaction in Linear Mixed‐Effect Models using the *lme* function. We also entered the subplot as a random effect term to account for repeated measures over months. In total, we had 4629 observations across 441 subplots. We also included a spatial structure using a Gaussian correlation structure (*corGaus*) and a spatial weights object based on distances in metres between pixels to control for spatial autocorrelation. To create the weights matrix, we converted the original latitude and longitude coordinates into a *SpatialPoints* object. These points were then transformed into the Universal Transverse Mercator (UTM) coordinate system, specific to our study area's UTM zone. Then, a *SpatialPointsDataFrame* object was created, combining the UTM coordinates with the data.

We performed stepwise model selection based on AIC rank using the *MuMIn* package. We employed the *dredge* function on each of our full models, ranking models based on their optimal balance between explanatory power and complexity (lower AIC). Subsequently, the best models were refitted based on the *dredge* output. We further explored the models' interactions with the *phia* package using the *testInteractions* function to compare the adjusted slopes for each season and evaluate the pairwise difference between the dry and wet seasons. Residual analysis was conducted through the examination of various diagnostic plots, including a residuals vs. fitted values plot, a histogram of residuals, and a normal quantile–quantile (Q–Q) plot. These visualisations aimed to assess the homoscedasticity, normality, and independence assumptions of the model. Additionally, scatter plots of normalised residuals against relevant covariates, main plots, and subplots were created to explore potential patterns or trends. Finally, we again assessed spatial autocorrelation in the models' residuals using Moran's *I* test. CV of bands and indices were log, square‐root, or cube‐root transformed to improve homoscedasticity of residuals. To further investigate the relationship between the predictor variable and the response variables, the *ggeffects* package was utilised to generate predictions from the model for SLA, considering random effects. The resulting predictions were plotted for the visual representation of the relationship between SLA and the coefficient of variation of the selected reflectance variables.

#### 2.4.4 Biome‐Wide SLA‐Reflectance Models (Amazon Basin)

To determine the universality of the relationship between SLA and CV of blue (intra‐month variation in the reflectance of the blue during the year 2019), we applied the same modelling approaches described above for another 130 Amazon plots with lower spatial resolution (100 × 100 m), located in 44 different regions. In addition, we included hydraulic variables, known for being related to sclerophylly, in the model to test their association with the variation in blue reflectance. For the Amazon, the variable CV of blue did not show a significant spatial autocorrelation, so we fitted a linear mixed‐effects model using the *lmer* function from the *lme4* package and tested the model residuals for spatial autocorrelation. The global model included SLA and the monthly means (across 2019–2022) of ESI, WUE, and Evap as predictor variables, all with interaction with season, with random effects of regions and individual plots—to account for repeated measures over the months. Finally, we used a simple linear model to compare the CWM of SLA across the 44 Amazon Regions. We also explored the relationships among SLA, latitude, longitude, ESI, WUE, and Evap with a PCA (Figure S5).

#### 2.4.5 Long‐Term Trends in Reflectance Stability

To investigate whether blue‐band reflectance stability has changed over the last four decades, we modelled trends in the coefficient of variation of blue (CV of blue) from Landsat for the 44 Amazon regions. The response variable was the seasonal CV of blue (within‐season variation in blue reflectance), derived from harmonised Landsat Collection 2 surface reflectance (30 m, 1984–2022) extracted in plot‐centred buffers around the 130 closed‐canopy primary forest plots. This design ensures that the trends represent functional changes in relatively undisturbed forests rather than a mixture of land‐use trajectories. A linear mixed‐effects model (LMM) was fitted using the *lmer* function in the *lme4* package in R. Fixed effects included year (continuous), season (wet vs. dry), region (44‐level factor) and their interaction (year × season × region). A random intercept for plot was included to account for repeated measurements through time within plots. Model selection, diagnostics, interaction tests and visualisation followed the same procedures described for the previous models. To evaluate whether the rate of change over time differs among regions, we examined the interaction between year and region, focusing on regional differences in the slope of year. The resulting Amazon‐wide maps (Section 2.4.6) represent the expected change in blue‐band reflectance stability conditional on forest being present, rather than a land‐cover‐agnostic trend surface.

#### 2.4.6 Changes in Reflectance Stability by Amazon Region

As a simple summary of the long‐term trends estimated in Section 2.4.5, we computed the percentage change in seasonal mean CV of blue (%Δ CV of blue) between two decadal periods, 1984–1993 and 2013–2022, for each plot and season. We then fitted a linear model using the *lm* function in R with %Δ CV of blue as the response and region, season and their interaction as predictors, using the same model selection and validation procedures as above. We used the *emmeans* package to obtain estimated marginal means (EMMs) for each region‐season combination and to test whether regional changes in CV within seasons differed significantly from zero. To produce spatially continuous maps, we projected regional predictions onto a 0.1° grid across the Amazon biome, using *sf* and *gstat* to create spatial objects and perform ordinary kriging with an exponential variogram model. These maps depict the interpolated spatial pattern of predicted changes in blue reflectance stability, interpreted as the expected trend in forested areas rather than across all land‐cover types.

#### 2.4.7 Linking Spectral Change to Climate Change

To assess the changes in climate and hydrology associated with the changes in variation of reflectance of the blue wavelength over the last four decades, we modelled the % Δ CV of blue between the periods 1984–1993 and 2013–2022 as a function of the climatic changes, characterised by the seasonal means of the variables from TerraClimate (Abatzoglou et al. 2018) between the two periods. To unravel the complex relationships between climatic variables, spatial positioning, and the changes in CV of blue, we started with exploring the relationships among spatial variables (easting and northing) and the % Δ of the climatic variables using Pearson's correlations (Figure S11). These key climatic and hydrological variables are intertwined, so a PCA was performed, normalising and centring them, to reduce dimensionality and capture the variance efficiently. The first three principal components were then integrated back into the dataset. We employed a linear mixed‐effects model (LMM) using the *lmer* function, specifying the % Δ CV of blue as the dependent variable and incorporating interactions between season and the principal components (PC1, PC2, and PC3), acknowledging the random effects by region. Model selection was performed as described before. This model was refitted and subjected to the same detailed analysis as the previous models. We tested for interactions between seasons and principal components using the *phia* package. Spatial autocorrelation in the residuals was assessed using Moran's *I* test, which showed no significant spatial autocorrelation (Moran *I* statistic = 0.0267, *p*‐value = 0.3637). We applied Ordinary Kriging as described above to interpolate the loadings of PCA 1 and PCA 2 across the Amazon, generating maps of spatial predictions and uncertainty estimates of climate change between the two periods.

## 3 Results

### 3.1 Does Reflectance Stability Increase With Sclerophylly?

Our methodology tests the link between sclerophylly and reflectance stability at two nested spatial scales: a fine‐scale, high‐resolution analysis leveraging ground‐truth vegetation and SLA data collected from subplots of 10 × 10 m in a Cerrado‐Amazon transition area, and a broader biome‐wide analysis from across the Amazon covering 130 sites for which community‐weighted means of SLA were obtained at an original spatial resolution of 100 × 100 m (Figure S1a and Table S1).

#### 3.1.1 High‐Resolution Analysis (Nova Xavantina)

Using Sentinel‐2 data and subplot‐level SLA in Nova Xavantina, we modelled the relationship between CV of reflectance and functional strategy. We fit a mixed effects model testing for the effect of SLA, season (dry and wet), and their interaction on the CV of vegetation reflectance. We selected the CV of the blue band (hereafter CV of blue) as the primary spectral predictor because it showed the strongest SLA association among the 14 candidates (Table S3; Figure S2a). In the dry season (*n* = 2644), the CV of blue‐SLA correlation was *r* = 0.342 (95% CI 0.308–0.376), single‐test *p* = 1.45 × 10^−73^, Holm‐adjusted *p* = 2.03 × 10^−72^, selection‐aware permutation *p* ≤ 0.0002 (0/5000 permutations), and Spearman *ρ* = 0.391 (*p* = 2.63 × 10^−97^). In the wet season (*n* = 2442), SLA‐CV associations were generally weak across bands/indices; the strongest association was red_edge4 (*r* = 0.111; 95% CI 0.071–0.150; single‐test *p* = 4.25 × 10^−8^; Holm‐adjusted *p* = 5.95 × 10^−7^; selection‐aware permutation *p* ≤ 0.0002; Spearman *ρ* = 0.106, *p* = 1.58 × 10^−7^; Table S3; Figure S2b).

**FIGURE 1:**
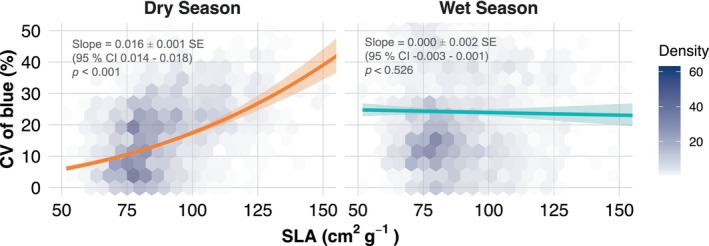
Relationship between community‐level Specific Leaf Area (SLA) and the intra‐month variation in reflectance of the blue wavelength (CV: Coefficient of Variation in %) during the wet and dry season of the year 2019 in Nova Xavantina, MT, Brazil. Each solid line represents the fitted values of a linear mixed‐effects model and the shaded area around them represents the 95% confidence intervals. The colour gradient within the hexagonal bins represents data density, with lighter shades indicating lower counts of data points and darker shades corresponding to higher counts.

Based on the model selection process (Table S4), the most effective model for explaining the variation in the blue band in the dataset included SLA, season and their interaction term (AIC = 11,402). The effect of SLA on CV of blue differed strongly between seasons (Dry‐Wet: *χ* ^2^(1) = 115.48, *p* < 0.001). In the dry season, SLA had a significantly positive effect on the cube‐root‐transformed CV of blue (slope_Dry = 0.016 ± 0.001 SE, 95% CI 0.014–0.018; *χ* ^2^(1) = 206.24, *p* < 0.001), whereas in the wet season the slope did not differ from zero (slope_Wet = 0.000 ± 0.001 SE, 95% CI −0.003 to 0.001; *χ* ^2^(1) = 0.40, *p* = 0.526). On the transformed scale, each additional 1 cm^2^ g^−1^ in SLA in the dry season increased the cube root of CV of blue by ~0.016 (*t* = 14.35, *p* < 0.001). To illustrate the effect on the original scale, we calculated marginal effects from the fitted model and plotted the back‐transformed predicted CV of blue across the observed SLA range (Figure 1).

We also found significant but weaker relationships (compared to SLA and CV of blue) between SLA and CV of green (AIC = 11288.8) for both the dry (Slope = 0.00, *χ* ^2^(1) = 106.50, *p* < 0.001) and wet season (Slope = 0.00, *χ* ^2^(1) = 27.07, *p* < 0.001); SLA and CV of NIR (AIC = 15743.5) also for both the dry (Slope = 0.01, *χ* ^2^(1) = 116.13, *p* < 0.001) and wet season (Slope = 0.01, *χ* ^2^(1) = 17.78, *p* < 0.001), and SLA and CV of red edge 4 regardless of season (AIC = 16041.4; Slope = 0.01, *t* = 8.52, *p* < 0.001; Figure S3). The Normalised Difference Red Edge index (NDRE) had no significant relationship with SLA (AIC = 17807.5).

#### 3.1.2 Biome‐Wide Analysis (Amazon Basin)

We extended the previous analysis to a biome‐wide scale, incorporating an assessment of the effect of plant water stress on reflectance stability. Using a simple linear model to compare the CWM of SLA across the 44 Amazon regions, our analysis revealed significant variability in SLA across regions (ANOVA *F*‐value = 10, df = 41, *p* < 0.001; Figure S4). The highest SLA value (lowest sclerophylly) was 174.11 cm^2^/g for sites in the Bolivia Cerro Pelao (CRP) region, with significant deviations from that in regions such as in Brazil Fazenda Santa Marta (SMT; −86.77, t = −10.28, *p* < 0.001), and Venezuela San Carlos de Rio Negro (SCR; −85.71, *t* = −8.29, *p* < 0.001). A Principal Component Analysis (PCA) including SLA, hydraulic variables and spatial variables (easting and northing) revealed distinct clustering by country (Figure S5). PC1, PC2, and PC3 (40.7%, 19.7%, and 16.8% explained variance respectively) displayed a spatial gradient across longitude and latitude on the measured variables, indicating increasing hydraulic stress and sclerophylly (lower SLA) towards the northern and eastern sites studied here. The clustering of points by country indicates distinct environmental or ecological conditions unique to each region. SLA showed a stronger positive association with Water Use Efficiency (WUE), although also positively associated with Evaporative Stress Index (ESI; Figure S6).

**FIGURE 2:**
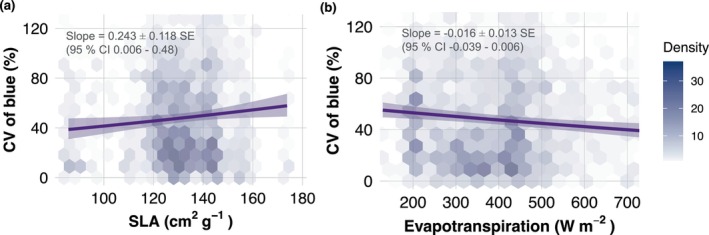
Effects of Specific Leaf Area (SLA) and instantaneous evapotranspiration on the intra‐month variation in reflectance of the blue wavelength (CV: Coefficient of Variation in %) during the year 2019 in the Amazon biome. (a) Effect of SLA on the CV of blue wavelength across seasons. (b) Effect of evapotranspiration on the CV of blue wavelength across seasons. Each solid line represents the fitted values of a linear mixed‐effects model and the shaded area around them represents the 95% confidence intervals. The colour gradient within the hexagonal bins represents data density, with lighter shades indicating lower counts of data points and darker shades corresponding to higher counts.

Again, using a linear mixed‐effects model testing the effect of SLA, season and this time also hydraulic variables on the CV of blue, we found that the best fitting model (AIC = 5971.7) included the factors SLA and evapotranspiration (Table S4). There was a significant positive effect of SLA on the CV of blue (Slope = 0.243 ± 0.118 SE, *t* = 2.16, 95% CI 0.006–0.48), while evapotranspiration exhibited a negative effect (Slope = −0.016 ± 0.011 SE, *t* = −1.44, 95% CI −0.039 to 0.006). Practically, each additional cm^2^/g in SLA was associated with an increase of 0.24% in the variation of blue (Figure 2a) and each additional W m^−2^ increase in evapotranspiration was associated with a decrease of 0.016% in the CV of blue (Figure 2b). The emergence of similar relationships between SLA and dry‐season variation in blue reflectance at both 10 m (Nova Xavantina) and 100 × 100 m (Amazon‐wide plots) indicates that our main findings are robust to moderate changes in the spatial resolution at which reflectance and traits are aggregated.

### 3.2 Has Leaf Sclerophylly Increased in the Amazon Over the Last Decades?

#### 3.2.1 Long‐Term Trends in Reflectance Stability

**FIGURE 3:**
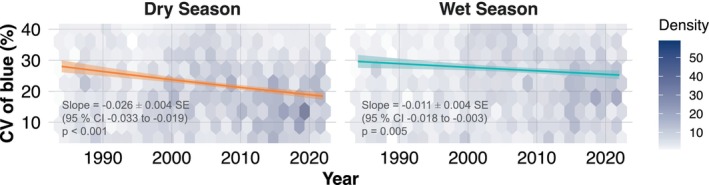
Predicted yearly trends in the Coefficient of Variation (CV in %) of reflectance at the blue wavelength by season over 39 years across 130 sites within the Amazon biome. The lines and shaded areas depict the model‐predicted values and their confidence intervals, respectively. Predictions and intervals have been back‐transformed to the original scale after model fitting. The colour gradient within the hexagonal bins represents data density, with lighter shades indicating lower counts of data points and darker shades corresponding to higher counts.

We assessed long‐term changes in reflectance stability using a linear mixed‐effects model that factored in variables such as year, season, and region. This model helped identify temporal trends and regional differences in rates of change as described below. The linear mixed model analysis indicated that CV of blue significantly varied over the years showing significant regional variations, with notable differences across seasons (AIC = 32205.7; Table S3). The dry season showed a significant decrease in CV of blue over the years (Slope = −0.026 ± 0.004 SE units per year, 95% CI −0.033 to −0.019, *χ* ^2^(1) = 49.91, *p* < 0.001), from about 28% in 1984 to about 18% in 2022, a reduction of roughly 10 percentage points (~34% relative decrease) over the 39 years (Figure 3a). In the wet season, the decrease was much less pronounced—around 5% (Slope = −0.011 ± 0.004 SE units per year, 95% CI −0.018 to −0.003, *χ* ^2^(1) = 8.00, *p* = 0.005; Figure 3b). These temporal trends are estimated while allowing for region‐specific deviations via the region × year and higher‐order interaction terms. Considering the region‐wide differences, only some regions exhibited significant year‐over‐year declines in within‐season variation of the blue reflectance: Bolivia Sacta (SCT; Slope = −0.10, *χ* ^2^(1) = 17.26, *p* < 0.01), Bolivia Las Londras (LSL; Slope = −0.07, *χ* ^2^(1) = 12.71, *p* < 0.05), Brazil BDFFP project (BDF; Slope = −0.06, *χ* ^2^(1) = 50.97, *p* < 0.001), Venezuela El Dorado (ELD; Slope = −0.10, *χ* ^2^(1) = 16.07, *p* < 0.01), and Venezuela Rio Grande (RIO; Slope = −0.22, *χ* ^2^(1) = 19.88, *p* < 0.001) in the dry season, and French Guiana Guyaflux (PAR; Slope = −0.08, *χ* ^2^(1) = 18.94, *p* < 0.01), and Peru Jenaro Herrera (JEN; Slope = −0.10, *χ* ^2^(1) = 22.01, *p* < 0.001) in the wet season (Figure S7).

#### 3.2.2 Changes in Reflectance Stability by Amazon Region

**FIGURE 4:**
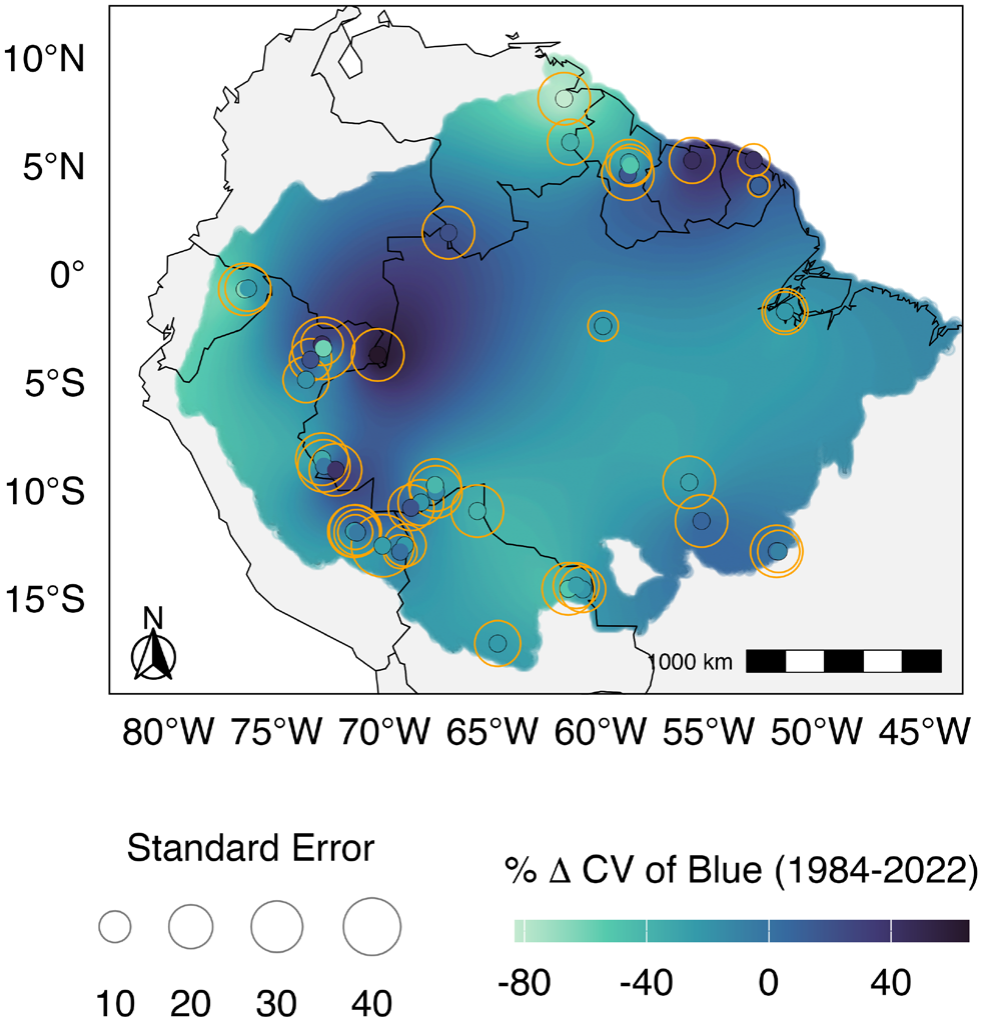
Predicted per cent change in the dry season variation of the blue reflectance (% Δ CV of Blue) between the periods 1984–1993 and 2013–2022 across regions of the Amazon biome. Points represent the study regions, with colours representing the model‐predicted percentage increase or decrease in CV of Blue between the two periods, and circle sizes representing the standard error around the predictions for each region. The continuous surface shows spatial predictions generated by Ordinary Kriging, and should be interpreted as the expected trend conditional on forest being present rather than across all land‐cover types. See Figure S9 for uncertainty levels. Map lines delineate study areas and do not necessarily depict accepted national boundaries.

We applied a simple linear model to assess the per cent change across Amazon regions in the within‐season variation of the blue reflectance (% Δ CV of blue), calculated as the change in the CV of blue between two 10‐year periods, 1984–1993 and 2013–2022. We found regional and seasonal variations in the % Δ CV of blue. The selected linear model revealed an interplay between regions, seasons, and their interaction in influencing the % Δ CV of blue between the two periods (AIC = 2086.2, *F*_79;128_ = 2.346, *p* < 0.001; Table S4). Several regions showed a significant % Δ CV of blue with differences between seasons (Figure 4 and Figure S8). In the dry season, significant per cent decreases in CV of blue were observed in Venezuela RIO (−82.942 ± 31.48, *p* = 0.009), Brazil BDF (−24.634 ± 9.49, *p* = 0.0105), Ecuador Bogi (BOG; −64.650 ± 31.48, *p* = 0.0420), Guyana Pibiri (PIB; −51.080 ± 18.17, *p* = 0.0057), Peru Manu (MNU; −24.968 ± 11.90, *p* = 0.0378), and Venezuela RIO (−82.942 ± 31.48, *p* = 0.0094); while we observed significant increases in Colombia Amacayacu Agua Pudre (AGP; 65.349 ± 31.48, *p* = 0.0399), French Guiana PAR (41.397 ± 10.49, *p* < 0.0001), and Peru Sucusari (SUC; 42.520 ± 18.17, *p* = 0.0208). In contrast, during the wet season, the significant results included per cent increases in CV of blue in Bolivia SCT (66.371 ± 22.26, *p* = 0.0034), Brazil BDF (44.572 ± 9.95, *p* < 0.0001), Brazil SMT (46.610 ± 18.17, *p* = 0.0115), Guyana Iwokrama (IWO; 61.622 ± 22.26, *p* = 0.0065), Peru Allpahuayo (ALP; 70.790 ± 18.17, *p* = 0.0002), and Venezuela SCR (76.105 ± 31.48, *p* = 0.0170). The regions Colombia Amacayacu: we excluded Lorena (LOR) and French Guiana Paracou (PAB) from the analysis due to a lack of spectral reflectance data for the earlier periods.

### 3.3 How Are Climatic Variables Correlated With the Observed Changes in Reflectance?

#### 3.3.1 Linking Spectral Change to Climate Change

**FIGURE 5:**
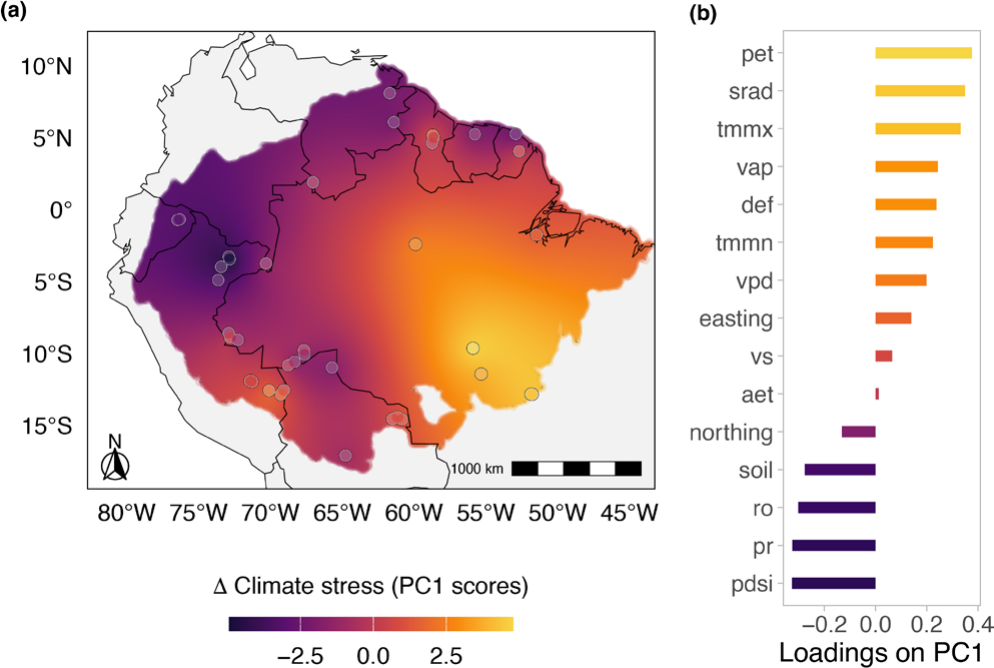
Changes in climatic conditions (Δ Climate stress) between the periods 1984–1993 and 2013–2022 in the Amazon biome. (a) location of the study regions of the Amazon biome (points), their average score (point colour) on the first axis of a Principal Component Analysis of climatic and hydrological factors (PC1; Figure S10), and predicted scores for the whole biome generated using interpolation by Ordinary Kriging (see Figure S9 for uncertainty levels). (b) loadings of each variable on PC1. Climatic and hydraulic variables from TerraClimate are the per cent variation in the annual mean between the periods 1984–1993 and 2013–2022 (See Table S5 for variable descriptions). aet = Actual Evapotranspiration; def = Climate Water Deficit; PDSI = Palmer Drought Severity Index; pet = Potential Evapotranspiration; pr = Precipitation; ro = Runoff; soil = Soil Moisture; srad = Downward Surface Shortwave Radiation; tmmn = Min Temperature; tmmx = Max Temperature; vap = Vapor Pressure; vpd = Vapor Pressure Deficit; vs. = Wind‐speed at 10 m; easting = Longitudinal degrees; northing = Latitudinal degrees. Map lines delineate study areas and do not necessarily depict accepted national boundaries.

To explore geographical gradients in climatic change over the period, we performed a Principal Component Analysis (PCA) on the climatic variable per cent variations over 39 years and spatial variables (easting and northing). The PCA indicates significant variances across regions in the Amazon. The first principal component (PC1), explaining 38.6% of the variance, is influenced positively by variables such as Δ pet (potential evapotranspiration), Δ srad (solar radiation), Δ tmmx (maximum temperature), Δ vap (vapour pressure), and Δ def (climate water deficit), and negatively by Δ PDSI (Palmer drought severity index—lower values indicate higher stress), Δ pr (precipitation), and Δ ro (runoff), suggesting that these factors contribute to climatic changes over the last decades (Figure S10). Thus, PC1 characterises a gradient of increasing climate stress over the years, which is spatially oriented from north to south and from west to east (Figure 5). The second principal component (PC2), accounting for 15.5% of the variance, shows substantial negative loadings from Δ aet (actual evapotranspiration), Δ tmmn (minimum temperature), and Δ vpd (vapour pressure deficit). A positive loading from easting suggests greater increases in those variables over the years in the western Amazon.

**FIGURE 6:**
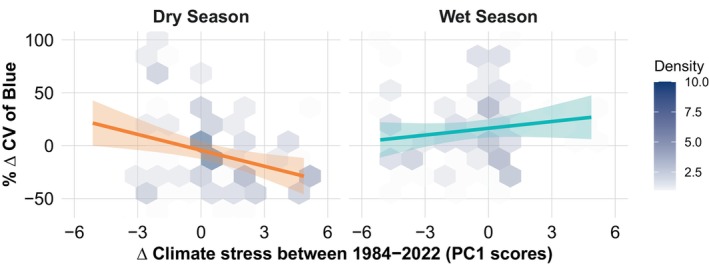
Relationship between the per cent variation of the blue reflectance (% Δ CV of Blue) and the change in climatic and hydrological conditions (Δ Climate stress) between the periods 1984–1993 and 2013–2022 in the Amazon biome. The first axis of the Principal Component Analysis (PCA) of climatic and hydrological changes over the period (PC1 scores) represents a gradient of increasing climatic stress over the last four decades at the Amazon sites (see Figures 5 and S10). The climatic and hydraulic variables used in the analysis are the per cent variation in the annual mean between the periods 1984–1993 and 2013–2022. See Table S5 for variable descriptions. The colour gradient within the hexagonal bins represents data density, with lighter shades indicating lower counts of data points and darker shades corresponding to higher counts.

We incorporated the first three axes of the PCA on the % Δ of the climatic and hydrological variables as predictors in a linear mixed‐effects model to assess their impact on the intra‐month variation of blue reflectance, considering the seasonal variations and spatial distributions across the Amazon. Our analysis revealed a significant association between the shifts in climate variables and the percent variation in CV of blue over 39 years. The best model (AIC = 2094.2) included the interaction term of PC1 (climate stress axis) and season, highlighting the varying effects of these variables across different seasons. The model demonstrated a significant positive effect of PC1 on Δ CV of blue in the dry season (Slope = −5.0176, *χ* ^2^(1) = 8.8393, *p* = 0.006), which was not significant in the wet season (Slope = 2.1347, *χ* ^2^(1) = 0.1944, *p* = 0.1943; Figure 6). This indicates that the change in the CV of blue during the dry season over the years was influenced by the changes in climatic and water availability, suggesting that decreases in the variation of blue were associated with increases in climatic stress, which in general increased from north to south and from west to east in the Amazon study sites. Spatial autocorrelation in the residuals was assessed using Moran's *I* test, which showed no significant spatial autocorrelation (Moran *I* statistic = 0.0267, *p*‐value = 0.3637).

## 4 Discussion

Our findings demonstrate that reflectance stability—particularly in the blue spectral band—is a sensitive indicator of ongoing functional change in the Amazon. Our approach included precise validation using ground‐truthing data, capturing details at nearly the individual tree crown level. In side‐by‐side plots in Nova Xavantina, we observed that vegetation characterised by greater sclerophylly exhibited higher reflectance stability during the dry season. This observation supports our hypothesis that vegetation at the conservative and slow end of the spectrum of plant economics (Díaz et al. 2016; Reich 2014; Wright et al. 2004) exhibits less variability in reflectance during the dry season. This stability likely arises from coordinated traits such as low leaf turnover, durable tissues, and synchronised phenology, which minimise seasonal shifts in pigment levels, water content, and light‐use efficiency (Chabot and Hicks 1982; Reich 2014; Westoby et al. 2002). In seasonally dry climates, these conservative strategies are expressed most strongly when water is limiting, so differences in drought‐response behaviour translate directly into differences in short‐term spectral variability: we expect conservative canopies to remain optically stable when soils are dry, whereas acquisitive canopies show stronger short‐term fluctuations in leaf water content, pigments and leaf area. By contrast, during the wet season, when soils are moist and most canopies are fully foliated and actively flushing leaves, short‐term variation in reflectance is larger overall and shared across strategies, so spectral variability is no longer tightly coupled to SLA. While SLA was used to calibrate this relationship, our aim was not to detect this trait directly via remote sensing. Instead, SLA served as an anchor for identifying vegetation with conservative strategies—those defined not by a single trait, but by an integrated syndrome of structural and phenological adaptations to drought. These adaptations are individually difficult to monitor at scale, but together they produce a detectable signal of reduced optical variability. To better understand the mechanisms behind this signal, future work should combine high‐resolution time series of temporally matched reflectance data with ground‐based monitoring of vegetation physiology and phenology.

By leveraging the full temporal depth of harmonised Landsat archives, we were able to detect a slow‐moving ecological trend: a ~35% basin‐wide increase in reflectance stability (a drop of ~10 percentage points in CV) over four decades, consistent with a shift toward more conservative vegetation strategies—particularly in increasingly water‐limited southern and eastern regions (Tavares et al. 2023). Based on our Sentinel‐2 calibration, this trend corresponds approximately to a reduction in SLA of 41.66 cm^2^/g—which simplistically extended suggests that within four decades the Amazon could exhibit SLA levels akin to the Amazon‐Cerrado transition zones, such as Nova Xavantina. However, we interpret this cautiously, as a proxy for a broader shift in plant economic strategies rather than SLA alone. Furthermore, conceptually, such functional shifts are consistent with scenarios in which increased sclerophylly and conservative strategies precede transitions to more open, fire‐prone ecosystems (Veldman 2016; Veldman and Putz 2011), but our data do not allow us to demonstrate such a structural trajectory empirically. The observed increase in reflectance stability integrates processes acting at intra‐annual scales, including phenotypic plasticity of canopy trees and adjustments in other traits associated with low variability in reflectance (e.g., leaf phenology, crown architecture, leaf angle, and canopy clumping) under increasing water and temperature stress. We therefore interpret our metric as an early signal of changing canopy function rather than direct evidence of a full structural transition towards a more open‐canopy state. If climate stress and disturbance regimes continue to intensify, these functional changes could still facilitate a shift towards more open ecosystems, characterised by heightened susceptibility to fires and reduced biomass, with potential declines in biodiversity, carbon storage and nutrient cycling (Boulton et al. 2022; Cano et al. 2022; Malhi et al. 2009). The implications of these changes extend beyond ecological concerns, such as impacts on fauna; ^43^ they could dramatically alter precipitation regimes, critically affecting agriculture and water supplies for hundreds of millions across South America (Staal et al. 2018). At the same time, some species may buffer these pressures by adjusting their physiology without large structural changes, which could support ecosystem resilience. Moreover, because our analysis is centred on closed‐canopy forest plots, the reflectance metric we develop primarily captures functional change within the tree canopy and does not explicitly resolve shifts in grasses and shrubs that would characterise a full structural transition toward a more open‐canopy state.

Crucially, the changes identified here were not homogeneous across the Amazon, with some regions showing lower or no change compared to others. This observation aligns with Tavares et al. (2023), who reported significant regional variations in tree hydraulic safety margins across the Amazon, suggesting diverse responses to environmental stressors. The variability in functional response is closely tied to anthropogenic impacts and the intensification of hydraulic stress in the Amazon. This suggests that anthropogenic disturbances, particularly land use and land cover changes, exacerbate local climate stress and this undermines the Amazon's resilience to climate change. The escalating deforestation rates lead to areas increasingly vulnerable to fires and biomass reduction, compromising the Amazon's structural integrity and its ability to regulate atmospheric carbon and moisture levels (Malhi et al. 2009; Sullivan et al. 2020; Tavares et al. 2023). Deforestation and burning practices have led to a loss of forest resilience and alterations in forest composition and structure (Boulton et al. 2022; van der Sande et al. 2023). These human‐induced changes are superimposed on climatic variations, creating a complex interplay of factors affecting the forest's health and stability. Our findings underscore the urgency for concerted conservation efforts and informed policy decisions to mitigate these changes. Reducing deforestation is crucial, as indicated by the amplified loss of resilience in areas closer to human activity (Lovejoy and Nobre 2019).

This study develops a systematic method for monitoring changes in vegetation reflectance patterns, providing insights into the resilience of tropical forests against environmental changes using remote sensing validated by field measurements. It introduces a method to measure functional traits of tropical forests, such as sclerophylly, indicative of transitions toward drought‐adapted vegetation facilitating planetary‐scale assessments of climate change impacts. This approach is crucial for developing technologies that aid in making informed decisions on biodiversity protection and climate adaptation (Kühl et al. 2020). Utilising satellite data from the past four decades we highlight change in the Amazon's response to climate change. However, the time frame might be too short to capture other long‐term tropical forest dynamics, such as the replacement of current adult individuals by others from more xerophytic species (Aleixo et al. 2019; Esquivel‐Muelbert et al. 2019). In addition, because our trait dataset focuses on sun‐leaf SLA and does not explicitly incorporate shade‐leaf traits or LAI/light‐extinction profiles, our results should be viewed as a first‐order approximation of canopy functional composition, and future work combining vertical trait profiles with canopy structure will be important to test robustness, particularly in more open systems. Even so, this relatively short‐term assessment already allows for the detection of changes potentially due to phenotypic plasticity in emergent trees, which are responsible for the reflectance captured by the satellites and which are particularly susceptible to hydraulic stress (Rao et al. 2019). These changes detected here serve as early warnings of climate stress and help evaluate the vulnerability of tropical forests to global changes at scale.

The observed trends in sclerophylly and their relation to climatic shifts have significant implications for Amazon‐forest resilience under climate change. A basin‐wide shift towards higher sclerophylly, inferred from a ~34% relative reduction in CV of blue since the 1980's, signals a strategic adjustment in multiple plant traits—longer leaf lifespans, conservative water use, synchronised phenology—that directly influence carbon and water cycling. Although these traits enhance drought resistance, they may also depress photosynthetic capacity and nutrient turnover, potentially lowering ecosystem productivity. Fundamentally, our results show that reflectance stability in the blue band provides an integrative, remotely sensed proxy for this conservative strategy, enabling continuous, large‐scale monitoring that complements traditional field surveys. By coupling long‐term satellite archives with ground data, the framework presented here allows early detection of functional reorganization, highlights vulnerable regions, and offers a scalable tool for guiding conservation and management as climate extremes intensify.

## Author Contributions

**Milton Barbosa:** conceptualization, data curation, formal analysis, funding acquisition, investigation, methodology, validation, visualization, writing – original draft, writing – review and editing. **Renata A. Maia:** data curation, formal analysis, investigation, visualization, writing – review and editing. **Imma Oliveras Menor:** data curation, funding acquisition, investigation, methodology, project administration, resources, writing – review and editing. **Ben Hur Marimon Junior:** data curation, funding acquisition, investigation, methodology, project administration, resources, writing – review and editing. **Beatriz Schwantes Marimon:** data curation, funding acquisition, investigation, methodology, project administration, resources, writing – review and editing. **G. Wilson Fernandes:** funding acquisition, investigation, project administration, resources, supervision, writing – review and editing. **Yadvinder Malhi:** data curation, funding acquisition, investigation, methodology, project administration, resources, supervision, writing – review and editing. **Jesús Aguirre‐Gutiérrez:** data curation, investigation, methodology, project administration, resources, supervision, validation, visualization, writing – review and editing.

## Funding

This work was supported by HORIZON EUROPE Marie Sklodowska‐Curie Actions, EP/Z003253/1; Conselho Nacional de Desenvolvimento Científico e Tecnológico, 403055/2022‐9; Leverhulme Trust, RPG‐2024‐342; Natural Environment Research Council (NERC), NE/T011084/1, NE/Z504191/1; Royal Society, RG/R1/251370.

## Conflicts of Interest

The authors declare no conflicts of interest.

## Supporting information


**Appendix S1:** gcb70727‐sup‐0001‐Pl.

## Data Availability

The plot‐ and miniplot‐level remote sensing and climate time series generated for this study are archived in Zenodo under the record “Data for: Detected shifts towards drought‐adaptive strategies in the Amazon Forest over the last four decades” (Barbosa et al. 2026), available at https://doi.org/10.5281/zenodo.18300311. The Google Earth Engine (GEE) scripts and R code used to derive and analyse these datasets are archived in Zenodo under “Code for: Detected shifts towards drought‐adaptive strategies in the Amazon Forest over the last four decades” (Barbosa 2026), https://doi.org/10.5281/zenodo.18268844, and mirrored at https://github.com/miltonbsjunior/barbosa_etal_2026_GCB_remore_sensing.git. The underlying satellite and climate products are publicly available from their original providers and were accessed as follows: Landsat Collection 2 Level‐2 Surface Reflectance for Landsat 5, 7 and 8 from the USGS EROS archive; Copernicus Sentinel‐2 Level‐2A (COPERNICUS/S2_SR) and the TerraClimate dataset (IDAHO_EPSCOR/TERRACLIMATE) via the Google Earth Engine data catalogue; and ECOSTRESS evapotranspiration products via NASA's AppEEARS tool (https://lpdaacsvc.cr.usgs.gov/appeears). Community‐weighted mean specific leaf area (CWM SLA) data were derived from previously published data in Aguirre‐Gutiérrez et al. (2025), as made available through Google Earth Engine. All other data are provided within the article and its Supporting Information or can be reproduced from the archived code and input datasets.
