# Supplementary material for: Detected Shifts Towards Drought‐Adaptive Strategies in the Amazon Forest Over the Last Four Decades
Source: Glob Chang Biol. 2026 Feb 4;32(2):e70727. doi: 10.1111/gcb.70727 (PMC12869351; doi:10.1111/gcb.70727)
Supplement: Supplementary file 1 — Appendix S1: gcb70727‐sup‐0001‐Pl. [file GCB-32-e70727-s001.pdf]

Supplementary Information for:

"Detected shifts towards drought-adaptive strategies in the Amazon Forest over the last four decades"

Milton Barbosa[1,2*], Renata A. Maia[1,2], Imma Oliveras Menor[3,6], Ben Hur Marimon Junior[4], Beatriz Schwantes Marimon[4], G. Wilson Fernandes[2,5], Yadvinder Malhi[6,7], Jesús Aguirre-Gutiérrez[1,7]

[1]Biodiversity and Earth Observation, Environmental Change Institute, School of Geography and the Environment, University of Oxford, United Kingdom.

[2]Department of Genetics, Ecology & Evolution, Federal University of Minas Gerais, Belo Horizonte, Brazil.

[3]AMAP (Botanique et Modélisation de l'Architecture des Plantes et des Végétations), Université de Montpellier, CIRAD, CNRS, INRAE IRD, Montpellier, France.

[4]Department of Biological Sciences, State University of Mato Grosso (UNEMAT), Nova Xavantina, Brazil.

[5]Knowledge Center for Biodiversity, 31270-901 Belo Horizonte, Brazil.

[6]Environmental Change Institute, School of Geography and the Environment, University of Oxford, United Kingdom.

[7]Leverhulme Centre for Nature Recovery, University of Oxford, United Kingdom

* Corresponding authors - Email: miltonbsjunior@gmail.com

Table S1 | Amazon Vegetation Sites. Approximate location and elevation of the 130 sites within the Amazon biome used in the study.

Country	Site code	Site Name	Elevation	Latitude	Longitude
Bolivia	Bolivia-CRP	Cerro Pelao	350	-14.54	-61.50
Bolivia	Bolivia-LFB	Los Fierros Bosque	245	-14.58	-60.83
Bolivia	Bolivia-LSL	Las Londras	190	-14.40	-61.14
Bolivia	Bolivia-RET	Reserva El Tigre	160	-10.97	-65.72
Bolivia	Bolivia-SCT	Sacta	248	-17.09	-64.77
Brazil	Brazil-AFL	Alta Floresta	275	-9.62	-55.89
Brazil	Brazil-BDF	BDFFP project	75	-2.34	-59.85
Brazil	Brazil-CAX	Caxiuana	15	-1.74	-51.46
Brazil	Brazil-DOI	RESEX Chico Mendes: Seringal Dois Irmãos	203	-10.55	-68.31
Brazil	Brazil-FEC	Fazenda Experimental Catuaba	204	-10.07	-67.62
Brazil	Brazil-FLO	Fazenda Floresta	377	-12.81	-51.85
Brazil	Brazil-MIN	Rio das Minas, Parque Nacional da Serra do Divisor	226	-8.57	-72.90
Brazil	Brazil-MTH	Marechal Thaumaturgo, Alto Rio Juruá	246	-8.88	-72.79
Brazil	Brazil-POR	RESEX Chico Mendes: Seringal Porongaba	268	-10.80	-68.77
Brazil	Brazil-RFH	Reserva Florestal Humaita	176	-9.75	-67.67
Brazil	Brazil-RST	Reserva Extrativista do Alto Juruá	279	-9.04	-72.27
Brazil	Brazil-SIP	Fazenda Continental, Sinop	385	-11.41	-55.32
Brazil	Brazil-SMT	Fazenda Santa Marta, Ribeirão Cascalheira	332	-12.82	-51.77
Brazil	Brazil-TEC	TEAM Caxiuana	15	-1.71	-51.43
Colombia	Colombia-AGP	Amacayacu: Agua Pudre	120	-3.72	-70.30
Colombia	Colombia-LOR	Amacayacu: Lorena	94	-3.06	-69.99
Ecuador	Ecuador-BOG	Bogi	284	-0.70	-76.47
Ecuador	Ecuador-TIP	Tiputini	250	-0.63	-76.14
French Guiana	French Guiana-NOU	Nouragues Petit Plateau	127	4.09	-52.67
French Guiana	French Guiana-PAB	Paracou	40	5.27	-52.92
French Guiana	French Guiana-PAR	Guyaflux	35	5.28	-52.92
Guyana	Guyana-FMH	Forest reserve Mabura	122	5.18	-58.69
Guyana	Guyana-IWO	Iwokrama	139	4.63	-58.72
Guyana	Guyana-PIB	Pibiri	94	5.03	-58.60
Peru	Peru-AGJ	Aguajal	300	-11.89	-71.36
Peru	Peru-ALP	Allpahuayo	130	-3.95	-73.43
Peru	Peru-BAR	Barranco	345	-11.90	-71.42
Peru	Peru-CUZ	Cuzco Amazonico	190	-12.53	-69.05
Peru	Peru-JEN	Jenaro Herrera	151	-4.88	-73.63
Peru	Peru-LAS	Los Amigos	280	-12.55	-70.09
Peru	Peru-MNU	Manu	358	-11.88	-71.21
Peru	Peru-PAK	Pakitza	347	-11.94	-71.27
Peru	Peru-SUC	Sucusari	118	-3.25	-72.89
Peru	Peru-TAM	Tambopata	225	-12.83	-69.26
Peru	Peru-YAN	Yanamono	132	-3.43	-72.84
Suriname	Suriname-KAB	Kabo	58	5.26	-55.77
Venezuela	Venezuela-ELD	El Dorado	404	6.11	-61.40
Venezuela	Venezuela-RIO	Rio Grande	318	8.11	-61.69
Venezuela	Venezuela-SCR	San Carlos de Rio Negro	105	1.93	-67.04

Table S2. Summary of mixed-effects models relating CV of blue reflectance to specific leaf area (SLA) and covariates, with and without explicit treatment of data availability (n_obs) and using a high-coverage subset (n_obs ≥ 3 per month). All models include random intercepts for *region* and *main_plot*. See Supplementary Methods.

Model	Dataset	Predictor	Estimate	SE	t value	AIC
M1	All site-months (2019)	Intercept	30.74	15.71	1.96	5971.7
		SLA	0.24	0.11	2.16	
		Evap	-0.02	0.01	-1.44	
M2	All site-months (2019), with n_obs	Intercept	12.83	15.76	0.81	5968.0
		n_obs	2.28	0.95	2.41	
		SLA	0.26	0.11	2.34	
M3	High-coverage subset (n_obs ≥ 3)	Intercept	29.59	16.06	1.84	4874.1
		Season (wet)	6.99	3.18	2.20	
		SLA	0.20	0.12	1.69	

Table S3 | Pearson correlation coefficients (*r*) between SLA and each reflectance band or vegetation index for the dry and wet seasons (2019). Reported p-values are two-sided and Holm-adjusted across 14 bands to account for multiple testing**.**

Band / Index	Pearson r (Dry)	Holm-adjusted p (Dry)	Pearson r (Wet)	Holm-adjusted p (Wet)
blue	0.342	2.03×10^{-72}	−0.010	1.00
red	0.295	2.66×10^{-53}	−0.011	1.00
green	0.276	1.68×10^{-46}	0.084	4.09×10^{-4}
nir	0.245	1.69×10^{-36}	0.074	3.05×10^{-3}
red_edge1	0.220	2.29×10^{-29}	0.040	2.89×10^{-1}
red_edge3	0.183	2.26×10^{-20}	0.073	3.07×10^{-3}
red_edge4	0.132	7.42×10^{-11}	0.111	5.95×10^{-7}
red_edge2	0.110	8.71×10^{-8}	0.077	1.70×10^{-3}
msavi	0.062	8.00×10^{-3}	−0.068	6.21×10^{-3}
ndvi	0.062	8.00×10^{-3}	−0.069	6.21×10^{-3}
evi	0.062	8.00×10^{-3}	−0.069	6.21×10^{-3}
ndre	−0.022	7.85×10^{-1}	−0.024	1.00
swir1	−0.022	7.85×10^{-1}	0.024	1.00
swir2	0.009	7.85×10^{-1}	0.017	1.00

Table S4 | Stepwise model selection. Models are ranked based on their optimal balance between explanatory power and complexity (lower AIC). CV = Coefficient of Variation; SLA = Specific Leaf Area; ESI = Evaporative Stress Index; WUE = Water Use Efficiency; Evap = Evapotranspiration.

**Nova Xavantina - Global model: CV of blue ~ sla : season + (1 | plot)**									
Model	(Intercept)	season	sla	season : sla	df	logLik	AIC	delta	weight
8.000	0.997	+	0.016	+	7	-5694.029	11402.059	0.000	1
4.000	1.751	+	0.007	NA	6	-5751.060	11514.120	112.061	4.64E-25
2.000	2.402	+	NA	NA	5	-5795.112	11600.223	198.164	9.31E-44
3.000	2.090	NA	0.007	NA	5	-5948.607	11907.215	505.156	2.03E-110
1.000	2.659	NA	NA	NA	4	-5980.436	11968.871	566.813	8.28E-124

**Amazon - Global model: CV of blue ~ sla : season + esi : season + evap : season + wue : season + (1 | region) + (1 | plot)**										
Model	(Intercept)	esi	evapotranspiration	sla	wue	df	logLik	AIC	delta	weight
11	30.742	NA	-0.016	0.243	NA	6	-2979.864	5971.728	0.000	0.084
9	25.031	NA	NA	0.239	NA	5	-2980.881	5971.762	0.033	0.082
12	17.936	15.450	-0.017	0.238	NA	7	-2979.643	5973.286	1.558	0.038
27	30.152	NA	-0.018	0.234	2.224	7	-2979.675	5973.351	1.622	0.037

**Amazon - Global model: CV of blue ~ year : season : region + (1 | plot)**										
Model	(Intercept)	year	region : year	season : year	region : season : year	df	logLik	AIC	delta	weight
128	56.041	-0.025	+	+	+	178	-15924.856	32205.713	0	0.999
64	14.209	-0.005	+	+	NA	135	-15974.914	32219.827	14.114	0.001
32	0.357	0.002	+	NA	NA	134	-15980.716	32229.432	23.720	7.06E-06
48	51.070	-0.023	NA	+	NA	92	-16028.632	32241.265	35.552	1.90E-08

**Amazon - Global model: % Δ CV of Blue ~ region + season + region : season**									
Model	(Intercept)	region	season	region : season	df	logLik	AIC	delta	weight
8	-51.291	+	+	+	81	-962.080	2086.160	0.000	0.999
3	-5.418	NA	+	NA	3	-1047.411	2100.821	14.661	0.001
1	4.181	NA	NA	NA	2	-1055.185	2114.370	28.210	7.48E-07
4	-31.669	+	+	NA	44	-1020.036	2128.072	41.912	7.92E-10
2	-19.521	+	NA	NA	43	-1032.007	2150.013	63.853	1.36E-14

Amazon - Global model : % Δ CV of Blue ~ PC1 : season + PC2 : season + PC3 : season + (1 | region)

Model	(Intercept)	PC1	PC2	PC3	season	PC1 : season	PC2 : season	PC3 : season	df	logLik	AIC	delta	weight
26	-4.363	-5.018	NA	NA	+	+	NA	NA	6	-1041.095	2094.2	0	0.283
30	-4.319	-4.934	NA	-3.062	+	+	NA	NA	7	-1040.142	2094.3	0.1	0.27
94	-4.475	-4.972	NA	-4.383	+	+	NA	+	8	-1039.82	2095.6	1.45	0.137
28	-4.416	-4.942	0.5619	NA	+	+	NA	NA	7	-1041.05	2096.1	1.91	0.109

Table S5 | Climatic and hydrological indicators obtained from TerraClimate.

Variable	Description	Units	Relevance to Study
aet (Actual Evapotranspiration)	Monthly total amount of water evaporated from the soil and transpired by plants	mm	Influences water availability for plant growth; changes in evapotranspiration affect vegetation water requirements
def (Climate Water Deficit)	Monthly total deficit between potential evapotranspiration and actual evapotranspiration	mm	Indicates water stress conditions; negative def values suggest drier conditions impacting vegetation
PDSI (Palmer Drought Severity Index)	Palmer drought severity index at the end of the month	unitless	Reflects drought severity; negative values indicate drier conditions potentially impacting vegetation health
pet (Potential Evapotranspiration)	Monthly total maximum amount of water that could be evaporated from the soil and transpired by plants	mm	Reflects the maximum evaporative demand under current climate conditions; changes may indicate shifts in climate patterns
pr (Precipitation)	Monthly total amount of precipitation, influencing water availability for plant growth	mm	Directly impacts water supply for vegetation; changes in precipitation patterns can impact plant health
ro (Runoff)	Monthly total amount of water that flows over the land surface	mm	Changes in runoff can affect soil moisture levels, influencing vegetation health
soil (Soil Moisture)	Total column soil moisture at the end of the month	mm	Soil properties influence nutrient availability and plant growth
srad (Downward Surface Shortwave Radiation)	Monthly total solar radiation reaching the Earth's surface, essential for photosynthesis	W/m^2	Directly impacts energy available for plant processes; changes in radiation affect vegetation growth
tmmn (Min Temperature)	Average minimum temperature for the month	°C	Temperature influences various aspects of plant metabolism and growth
tmmx (Max Temperature)	Average maximum temperature for the month	°C	Higher temperatures can influence plant physiology and growth
vap (Vapor Pressure)	Average vapour pressure for the month, related to humidity	kPa	Influences plant transpiration; reflects atmospheric moisture conditions
vpd (Vapor Pressure Deficit)	Average vapour pressure deficit for the month	kPa	Reflects atmospheric drying conditions; affects plant water loss
vs (Wind-speed at 10m)	Average wind speed for the month	m/s	Influences various ecological processes, including seed dispersal and plant responses to mechanical stress

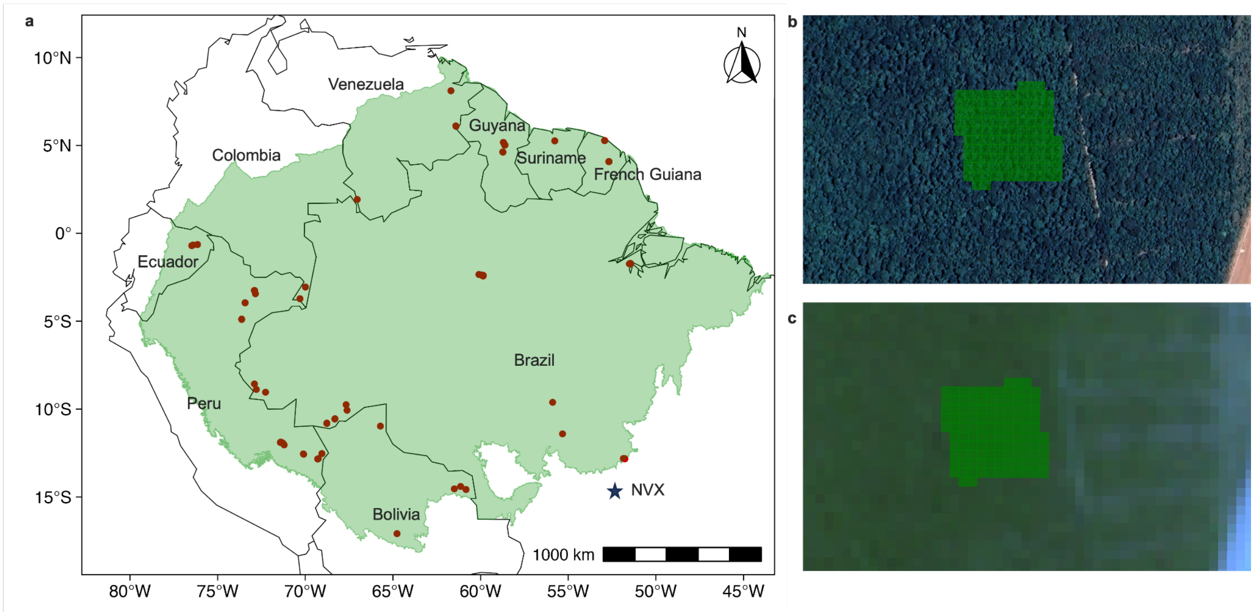

Fig. S1 | Location of the study sites and overlap of the subplots with the satellite pixels. **a,** Nova Xavantina plots (star), in a Cerrado-Amazon transition region in Brazil, and the Amazon vegetation sites (dots), spanning nine countries. **b,** the Nova Xavantina 'subplots' of 10 x 10 m, which were the unit used to calculate the trait community weighted mean based on the crown area of the trees in that pixel; and **c**, pixels of the Copernicus Sentinel-2 satellite for bands with native 10 m resolution showing the geospatial matching with the forest subplots. Map lines delineate study areas and do not necessarily depict accepted national boundaries.

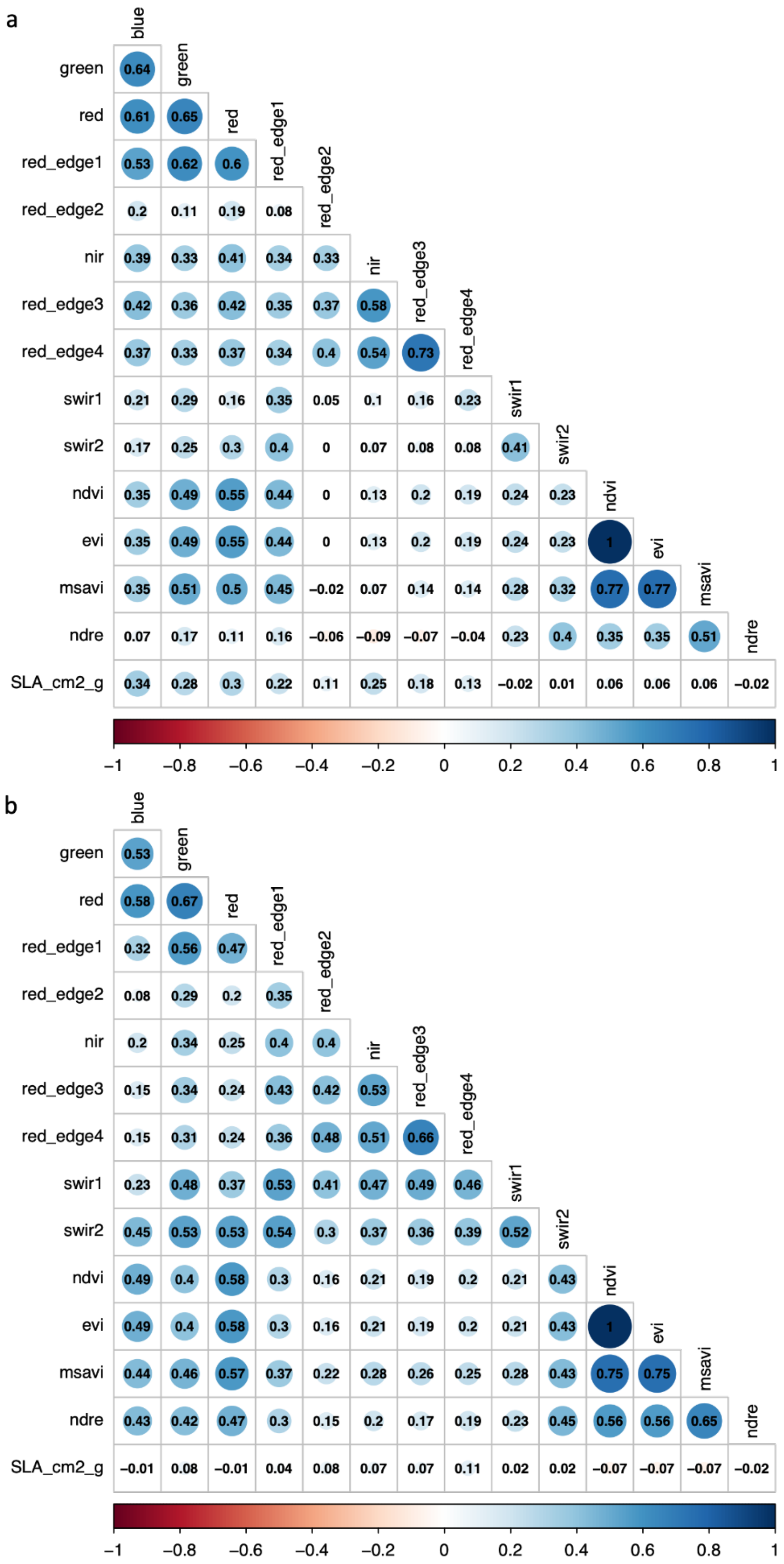

Fig. S2 | Pearson Correlation coefficients among the trait and reflectance variables in Nova Xavantina, Brazil during the (a) dry and (b) wet seasons. Reflectance variables are the intra-month coefficients of variation (%) over the year 2019. Asat = photosynthetic capacity at light saturated carbon assimilation rates (μmol m^{-2} s^{-1}); Amax: photosynthetic capacity at maximum carbon assimilation rates (μmol m^{-2} s^{-1}).

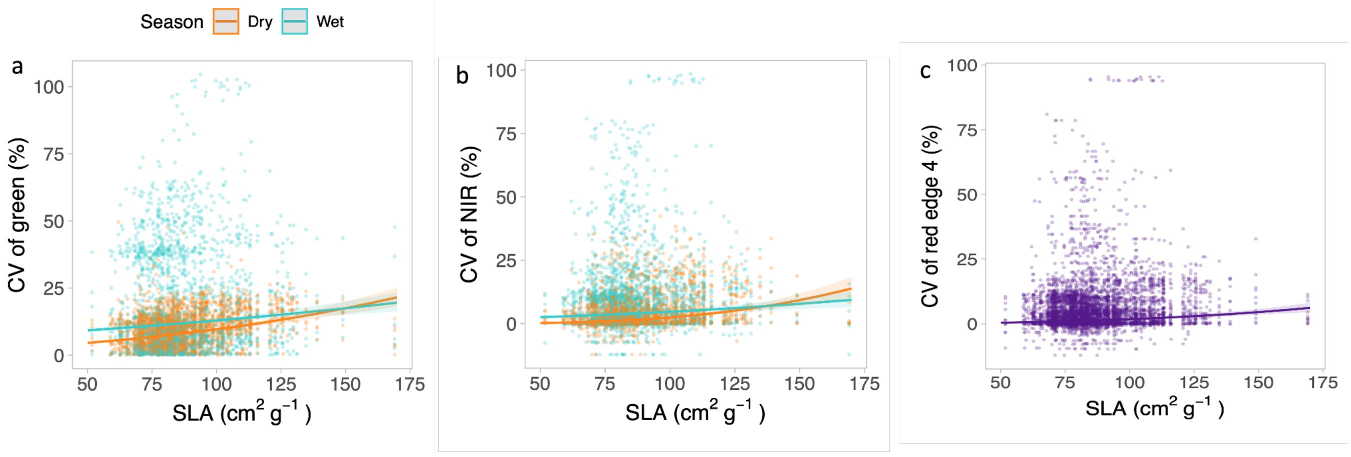

Fig. S3 | Conditional effects of Specific Leaf Area (SLA, in cm^2 per g) on the intra-month variation in reflectance (CV: Coefficient of Variation in %) of the (a) green and (b) NIR wavelengths across different seasons, and (c) red edge 4 band regardless of the season in Nova Xavantina, MT, Brazil. Each line represents the fitted values of a linear mixed-effects model.

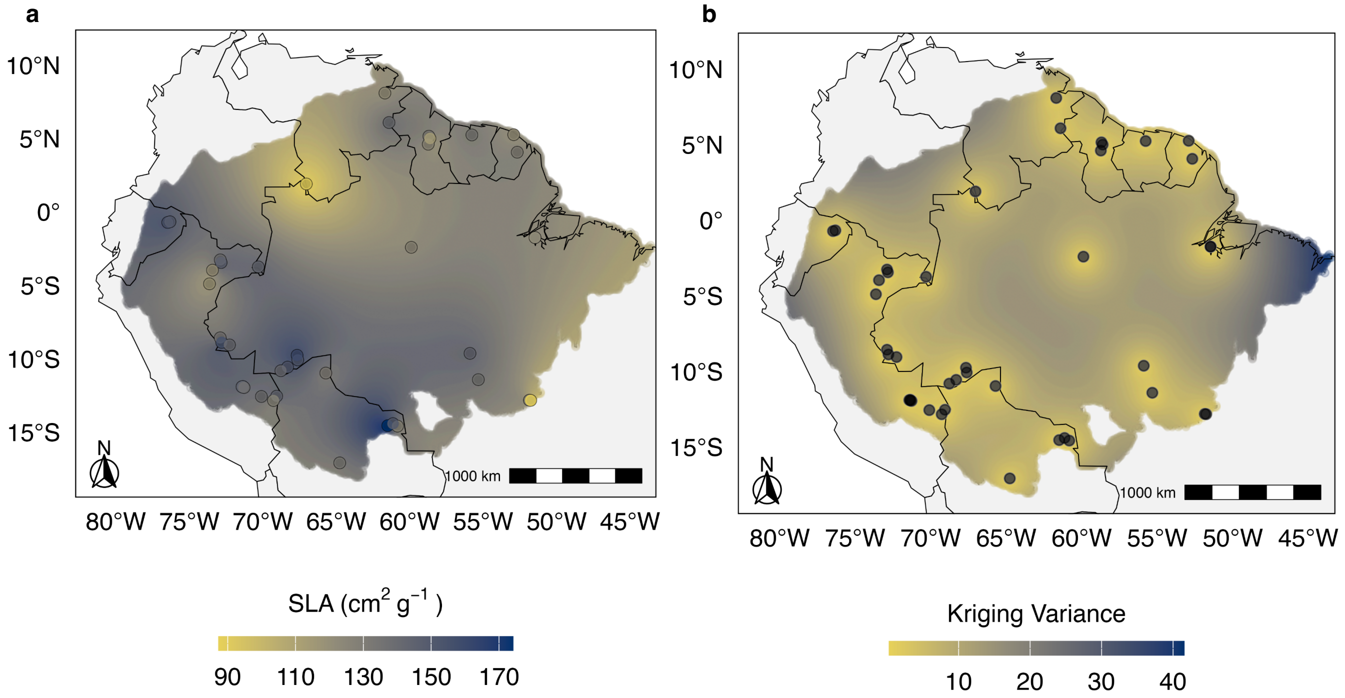

Fig. S4 | Specific Leaf Area (SLA) for regions of the Amazon biome. a, average Community-Weighted Mean of SLA (colour) for each study region (point) and predictions for the whole biome generated using interpolation by Ordinary Kriging. The interpolated surface should be interpreted as the expected SLA for forested areas rather than across all land-cover types. **b,** study regions (points) and uncertainty level of interpolated predictions. Map lines delineate study areas and do not necessarily depict accepted national boundaries.

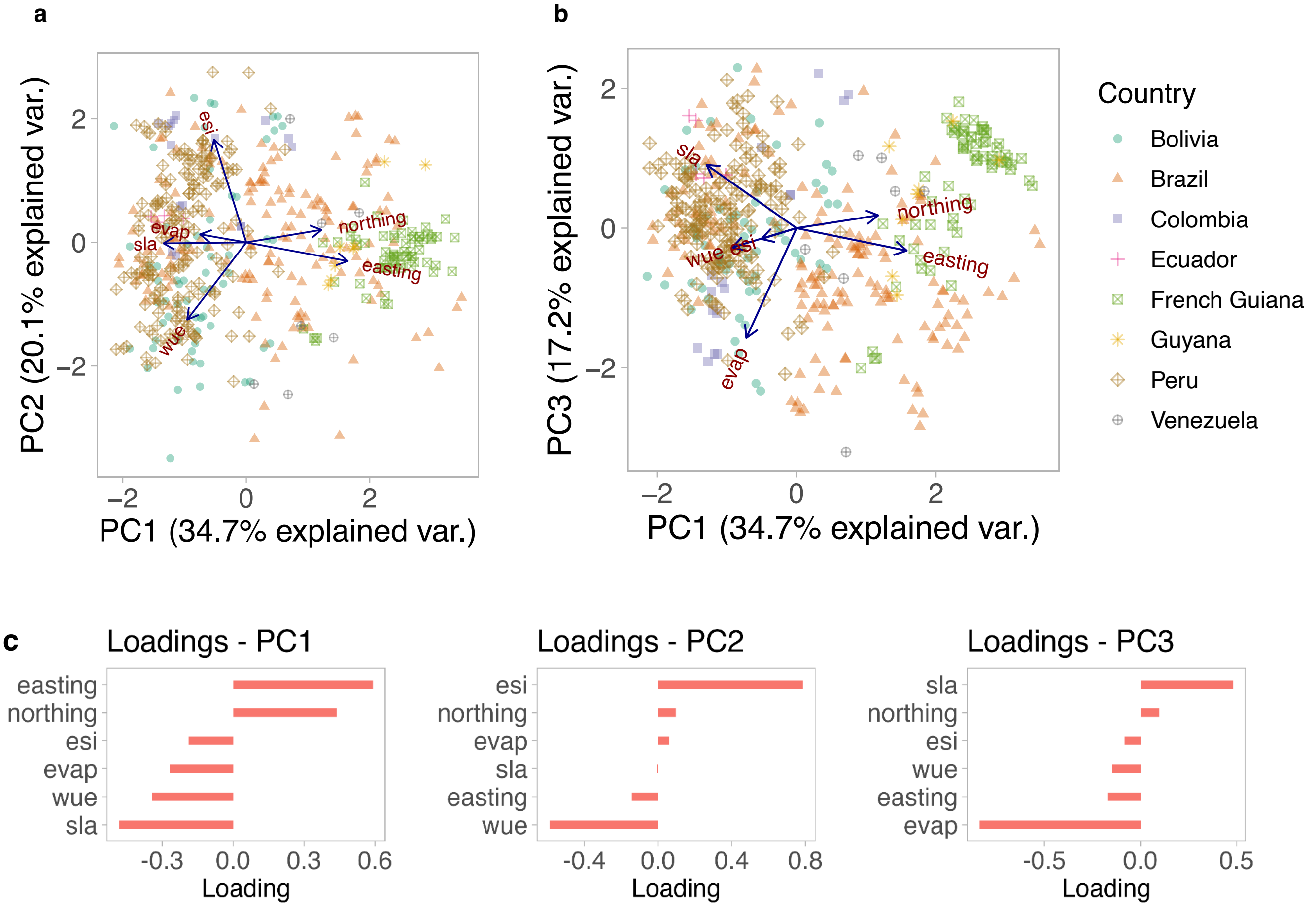

Fig. S5 | First and second (a) and first and third (b) axis, and loadings (c) of a Principal Component Analysis (PCA) calculated on normalised variables for Amazon sites. SLA = Specific Leaf Area; WUE = Water Use Efficiency; ESI = Evaporative Stress Index (high values mean low stress); Evap = Instantaneous Evapotranspiration; easting = Longitudinal degrees; and northing = Latitudinal degrees. Hydraulic variables are the month means over the years 2019-2022.

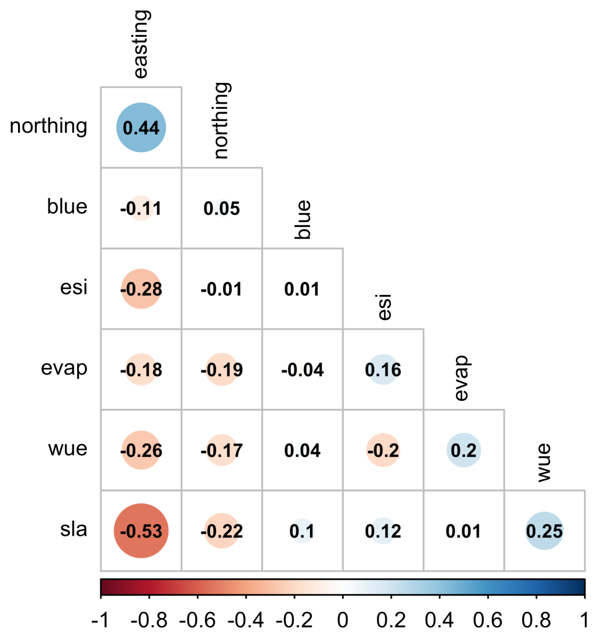

Fig. S6 | Pearson Correlation coefficients between SLA, spectral, and hydraulic variables in 130 Amazon sites. SLA= Specific Leaf Area; WUE = Water Use Efficiency; ESI = Evaporative Stress Index; Evap = Instantaneous Evapotranspiration; Easting = Longitudinal degrees; and Northing = Latitudinal degrees; blue = intra-month coefficient of variation (CV in %) for the year 2019. Hydraulic variables are the month means across the years 2019-2022.

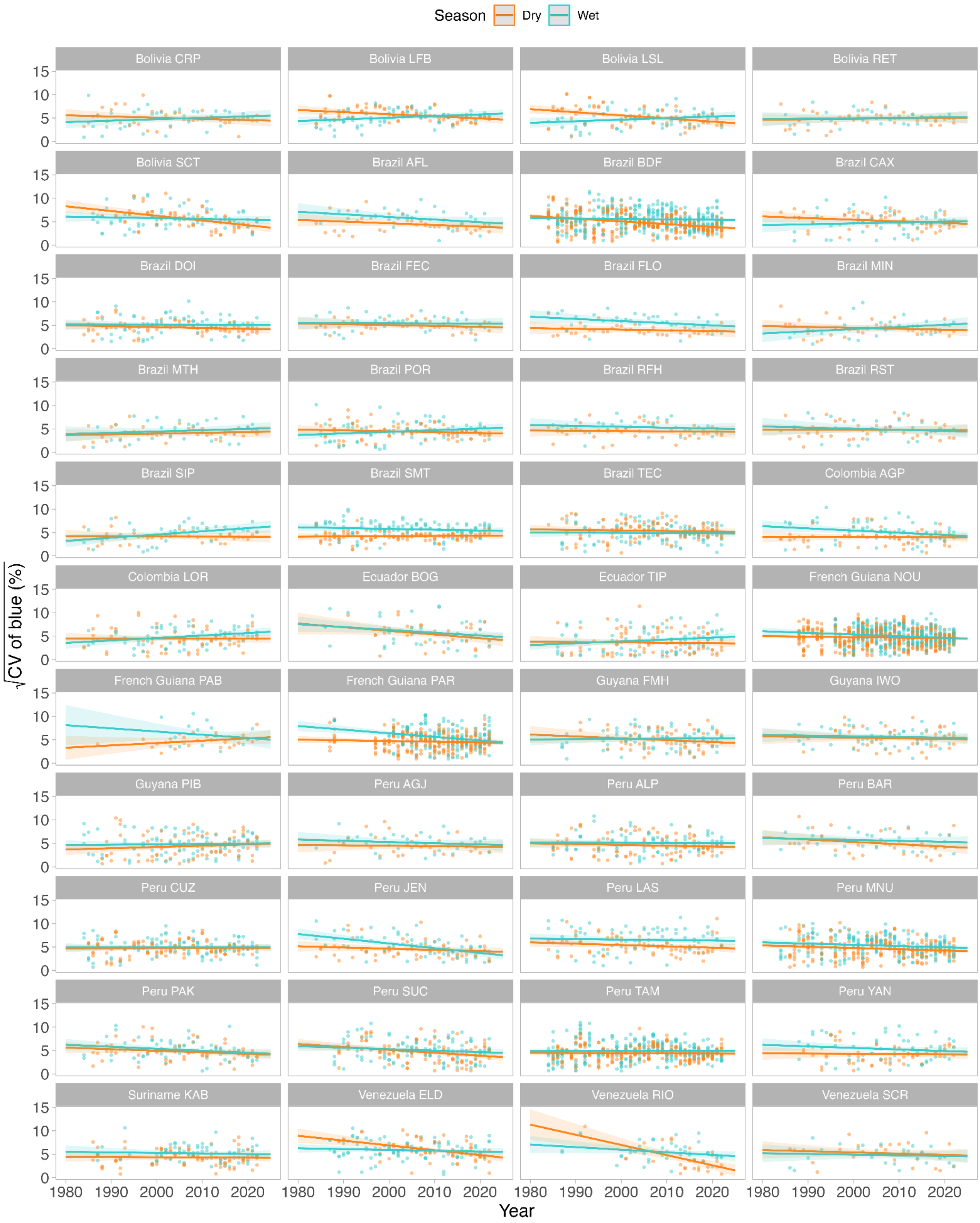

Fig. S7 | Predicted Yearly Trends in the Coefficient of Variation (CV in %) of reflectance at the blue wavelength by season and regions of the Amazon biome. Each dot represents a data point for a particular plot in the Amazon Forest. The lines and shaded areas depict the model-predicted values and their confidence intervals, respectively. Predictions and intervals have not been back-transformed and are presented in square root scale.

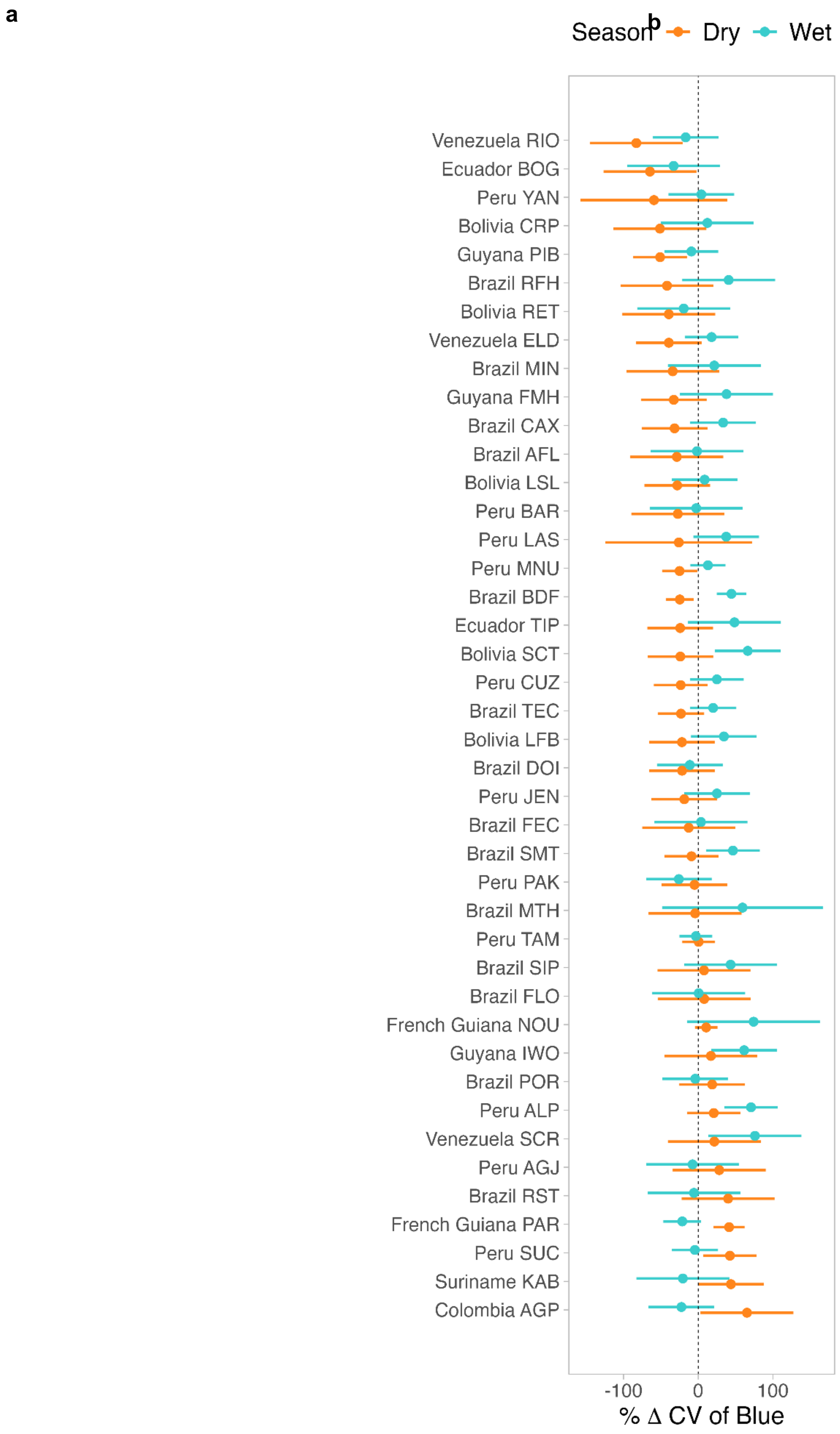

Fig. S8 | Predicted per cent change in the within-season variation of the blue reflectance (% Δ CV of Blue) between the periods 1984-1993 and 2013-2022 by season and region of the Amazon biome. Each point and line represent a model-predicted value and their confidence intervals, respectively.

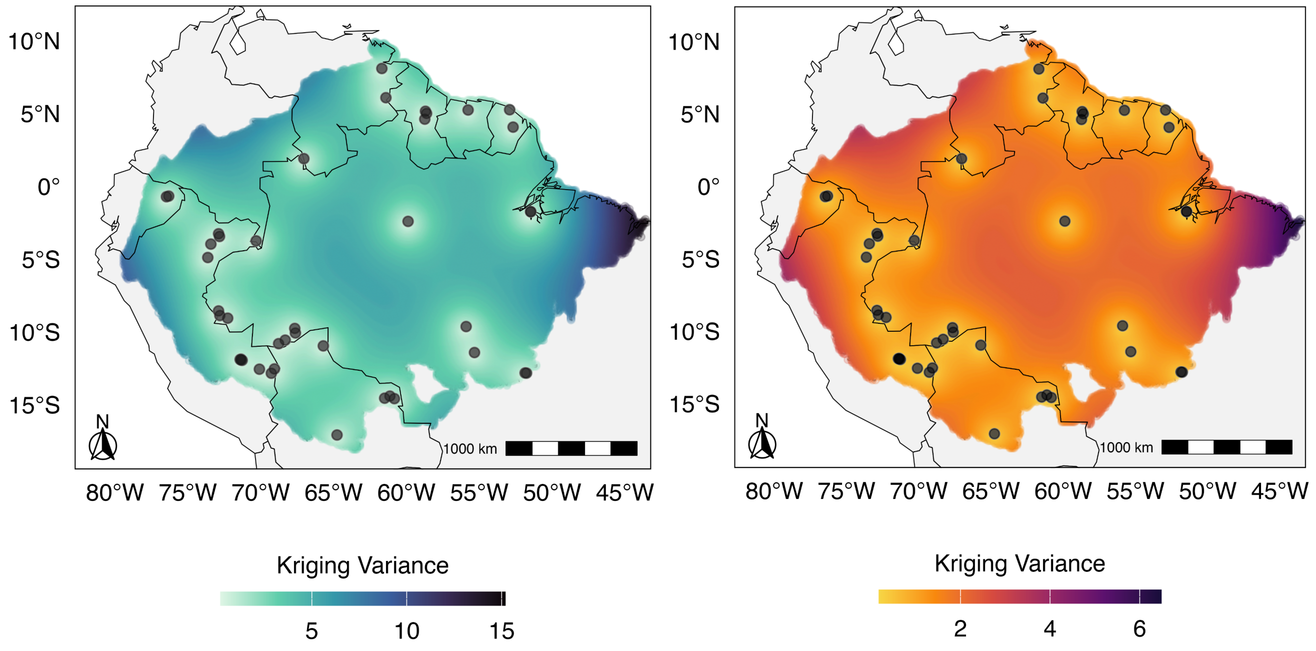

Fig. S9 | Uncertainty levels of interpolated predictions using Ordinary Kriging. **a,** uncertainty around the predictions of per cent change in the dry season variation of the blue reflectance (% Δ CV of Blue) between the periods 1984-1993 and 2013-2022 across regions of the Amazon biome (See Fig. 4 for the actual predictions). **b,** uncertainty around the predictions of change in climatic conditions (Δ Climate stress) between the periods 1984-1993 and 2013-2022 in the Amazon biome (See Fig. 5 for the actual predictions). Points represent the study regions, with colours representing the Kriging variance. Map lines delineate study areas and do not necessarily depict accepted national boundaries.

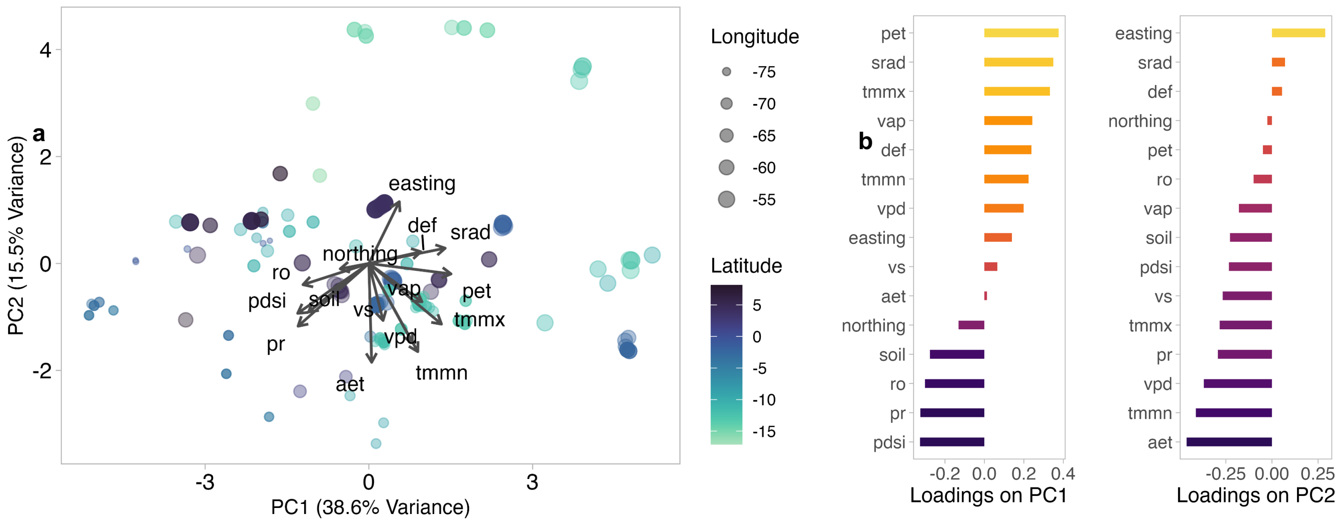

Fig. S10 | Principal Component Analysis (PCA) of changes in climatic and hydrological factors between the periods 1984-1993 and 2013-2022 in the Amazon biome. a, First and second axes of a Principal Component Analysis (PCA) on the per cent change (%Δ) of climatic and hydrological factors for the Amazon sites. Each point represents a site in the Amazon biome, colour-coded by latitude and size-coded by longitude. **b**, loadings of PC1 and PC2 of each climatic and hydraulic variable from TerraClimate, which are their per cent variation in the annual

mean between the periods 1984-1993 and 2013-2022 (See Table S3 for variable descriptions). aet = Actual Evapotranspiration; def = Climate Water Deficit; PDSI = Palmer Drought Severity Index; pet = Potential Evapotranspiration; pr = Precipitation; ro = Runoff; soil = Soil Moisture; srad = Downward Surface Shortwave Radiation; tmmn = Min Temperature; tmmx = Max Temperature; vap = Vapor Pressure; vpd = Vapor Pressure Deficit; vs = Wind-speed at 10m; easting = Longitudinal degrees; northing = Latitudinal degrees.

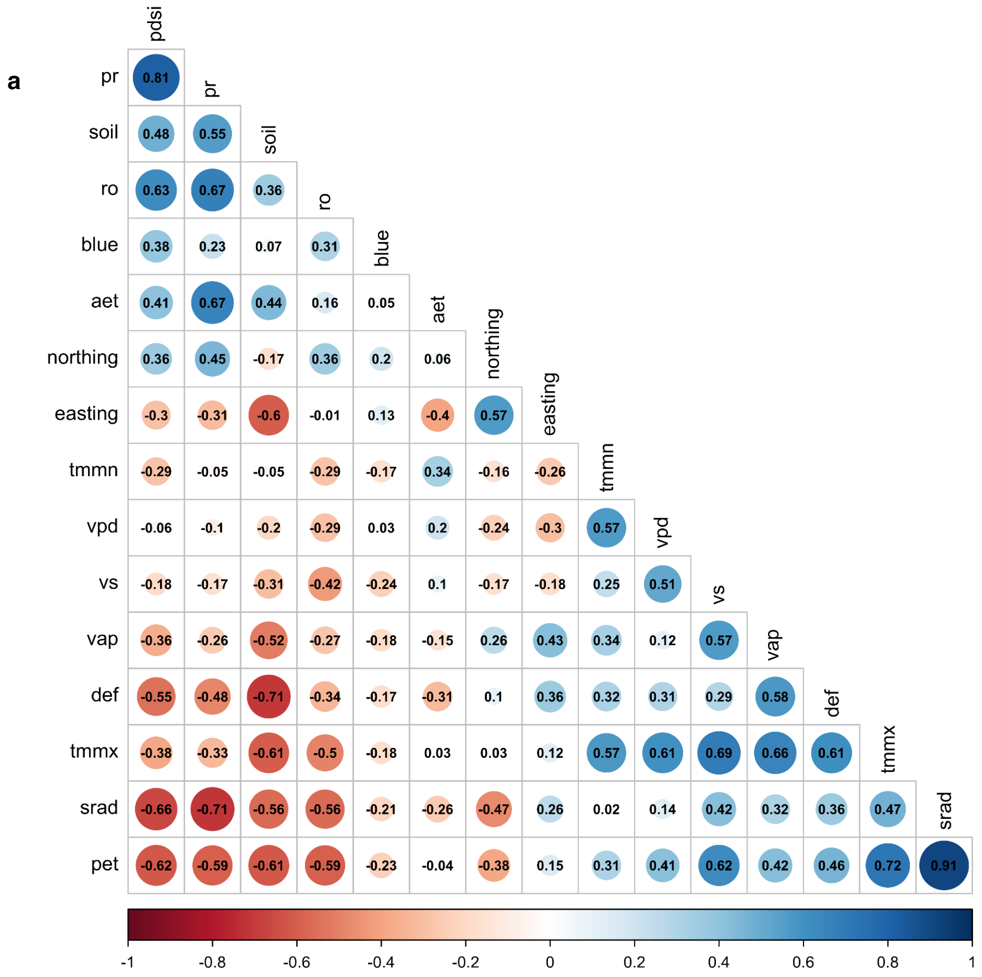

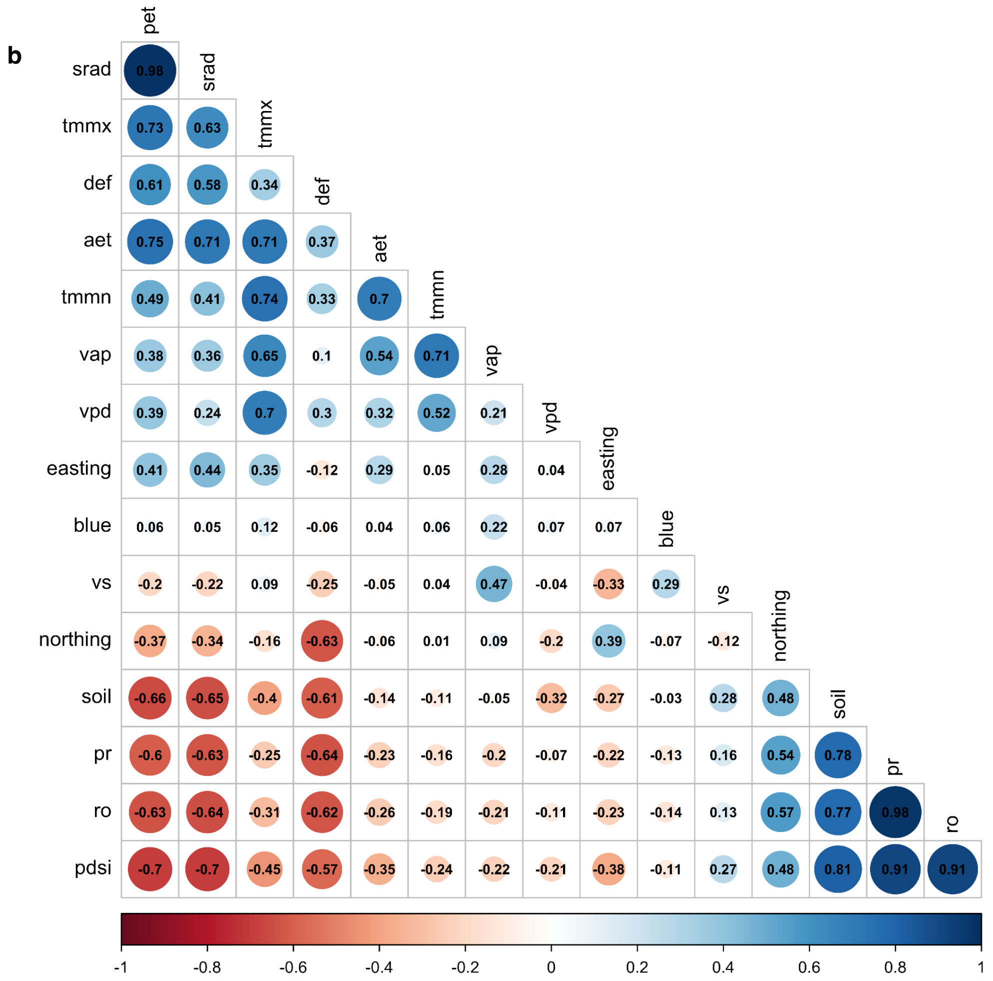

Fig. S11 | Pearson Correlation coefficients between the delta variation in the season mean of spectral, climatic, and hydraulic variables between the periods 1984-1993 and 2013-2022 in Amazon sites, during the (a) dry and (b) wet seasons. aet = Actual Evapotranspiration; def = Climate Water Deficit; PDSI = Palmer Drought Severity Index; pet = Potential Evapotranspiration; pr = Precipitation; ro = Runoff; soil = Soil Moisture; srad = Downward Surface Shortwave Radiation; tmmn = Min Temperature; tmmx = Max Temperature; vap = Vapor Pressure; vpd = Vapor Pressure Deficit; vs = Wind-speed at 10m; easting = Longitudinal degrees; northing = Latitudinal degrees. See Table S2 for variable descriptions.

Supplementary Methods

1 Processing of satellite reflectance and vegetation index time series

This section describes the acquisition, preprocessing, correction, and harmonisation of the satellite reflectance products used in this study, including cross-sensor spatial standardisation, cloud and quality masking, atmospheric/surface-reflectance corrections, and the derivation of vegetation indices and time-series metrics. All processing was implemented in Google Earth Engine (GEE) using custom scripts. All processed remote sensing

and climate time series, together with the full Google Earth Engine workflows, are archived in Zenodo (Barbosa, 2026; Barbosa et al., 2026).

1.1 Sampling locations and spatial units

All satellite-based analyses were extracted for fixed geographic units defined by longitude-latitude coordinates and associated polygons.

***1.1.1 Nova Xavantina (pixel-aligned subplots)* -** Field sampling was designed to match the 10 m native resolution of Sentinel-2 surface reflectance. Following Aguirre-Gutiérrez et al. (2021), permanent plots were subdivided into contiguous 10 x 10 m subplots, each intended to correspond to a single Sentinel-2 pixel. Plot boundaries were surveyed using differential GPS (DGPS), providing sub-meter accuracy for all corner coordinates. The subplot grid was constructed directly from these DGPS points and overlaid on Sentinel-2 imagery, enabling one-to-one matching between subplots and pixels without spatial resampling for most spectral bands (Fig. S1b and c). Individual trees were geolocated in the same DGPS reference frame. Where crown mapping was not available, crown dimensions were estimated using regionally calibrated allometric equations anchored to DGPS-referenced stem coordinates. Functional trait values were aggregated at the subplot level as community-weighted means, weighting each species by its proportional crown area within the corresponding 10 x 10 m unit.

***1.1.2 Regional and pan-Amazon analyses (plot buffers)* -** For broader-scale analyses (100 × 100 m field plots), plot coordinates do not generally align with the Sentinel-2 grid. We therefore buffered each plot centre by 50 m and converted it to a square polygon, within which all valid Sentinel-2 pixels were averaged to obtain plot-level reflectance and index time series.

1.2 Sentinel-2 surface reflectance data

Satellite reflectance and vegetation indices were derived from Sentinel-2 Level-2A surface reflectance (COPERNICUS/S2_SR) for the period 1 January 2019 to 31 December 2022. All images intersecting the polygon collection (buffer-based mini-plots or pixel-aligned subplots) were retained. Clouds, shadows and snow were removed using a combined masking strategy: cloud probability <5% (MSK_CLDPRB), snow probability <5% (MSK_SNWPRB), and exclusion of pixels classified as cloud shadow (SCL class 3) or cirrus (SCL class 10). The mask was applied uniformly prior to all band and index calculations.

1.2.1 Spectral band selection and preprocessing - From each cloud-free image, we extracted surface reflectance for bands B2, B3, B4 and B8 at 10 m native resolution (blue, green, red, NIR) and bands B5, B6, B7, B8A, B11 and B12 at 20 m native resolution (red_edge1, red_edge2, red_edge3, red_edge4, swir1, swir2). For processing in Google Earth Engine, all bands were mapped onto a common 10 m grid so that they could be stacked and extracted for the same set of polygons; upsampling of 20 m bands in this step does not add independent spatial information, and the effective spatial support of analyses involving red-edge and SWIR bands remains constrained by their

original 20 m resolution. Internally, bands were standardised to the names: blue, green, red, red_edge1, red_edge2, red_edge3, nir, red_edge4, swir1 and swir2.

1.2.2 Vegetation and moisture indices - Five spectral indices were calculated from masked reflectance: NDVI: red and near-infrared; EVI: near-infrared, red and blue; MSAVI: soil-adjusted vegetation index; NDMI: near-infrared and SWIR1; NDRE: red-edge (B8A) and near-infrared. Only valid (unmasked) pixels contributed to index values.

1.2.3 Extraction of plot-level and pixel-level time series - For each Sentinel-2 acquisition, mean reflectance and index values were extracted for each spatial unit using `reduceRegions` at 10 m scale. At Nova Xavantina, each extraction corresponds to a single 10 x 10 m pixel/subplot; for buffer-based plots (100 x 100m), values represent the mean across all valid pixels intersecting the polygon. Observations with no valid pixels on a given date were recorded as NoData and excluded from subsequent analyses. Monthly CVs of blue reflectance were computed only for plot-month combinations with ≥2 valid acquisitions (cloud/shadow/snow-free; see 1.2), and for the Amazon-wide Sentinel-2 analysis we quantified the pattern of missing CVs and its potential impact on the SLA-CV(blue) relationship (Table S2; Section 1.3).

1.3 Treatment of missing data and data-availability diagnostics

Sentinel-2 time series contain gaps due to cloud/shadow masking and other low-quality observations (Section 1.2). To assess whether uneven sampling biases our temporal stability metrics, we implemented the following diagnostics and robustness checks.

1.3.1 Data availability and CV computation - For each plot-month and spectral variable, CV (%, within-month) was computed only when ≥2 valid observations were available, ensuring a minimum temporal basis for estimating variability. Data availability varied seasonally as expected, with the highest coverage during the mid-year dry season. In 2019, the ≥2-observation criterion was met in 82.3% of plot-month combinations, and 60.2% had ≥3 valid observations; all months retained non-zero coverage at most sites (Figs. S12-S13).

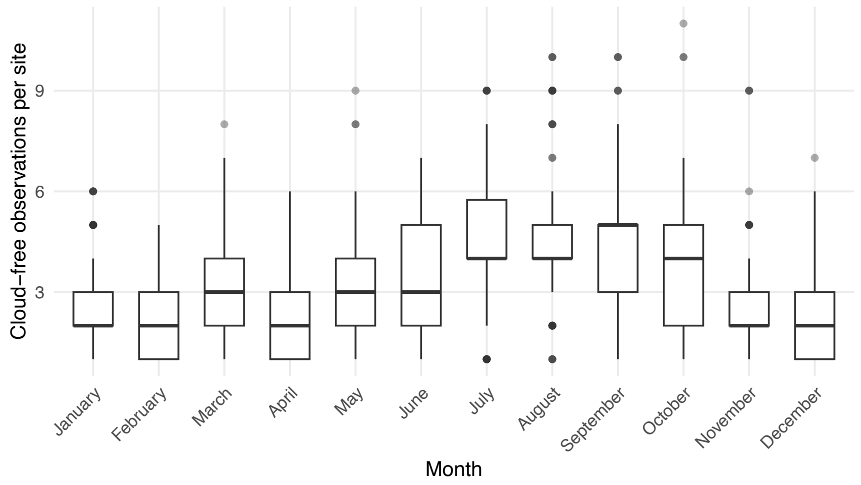

Figure S12 | Distribution of the number of valid (cloud- and shadow-free) Sentinel-2 observations per site in 2019 across Amazon basin study plots. Although data availability varies seasonally, all months retain non-zero coverage at most sites, and the core dry-season months receive the highest numbers of observations.

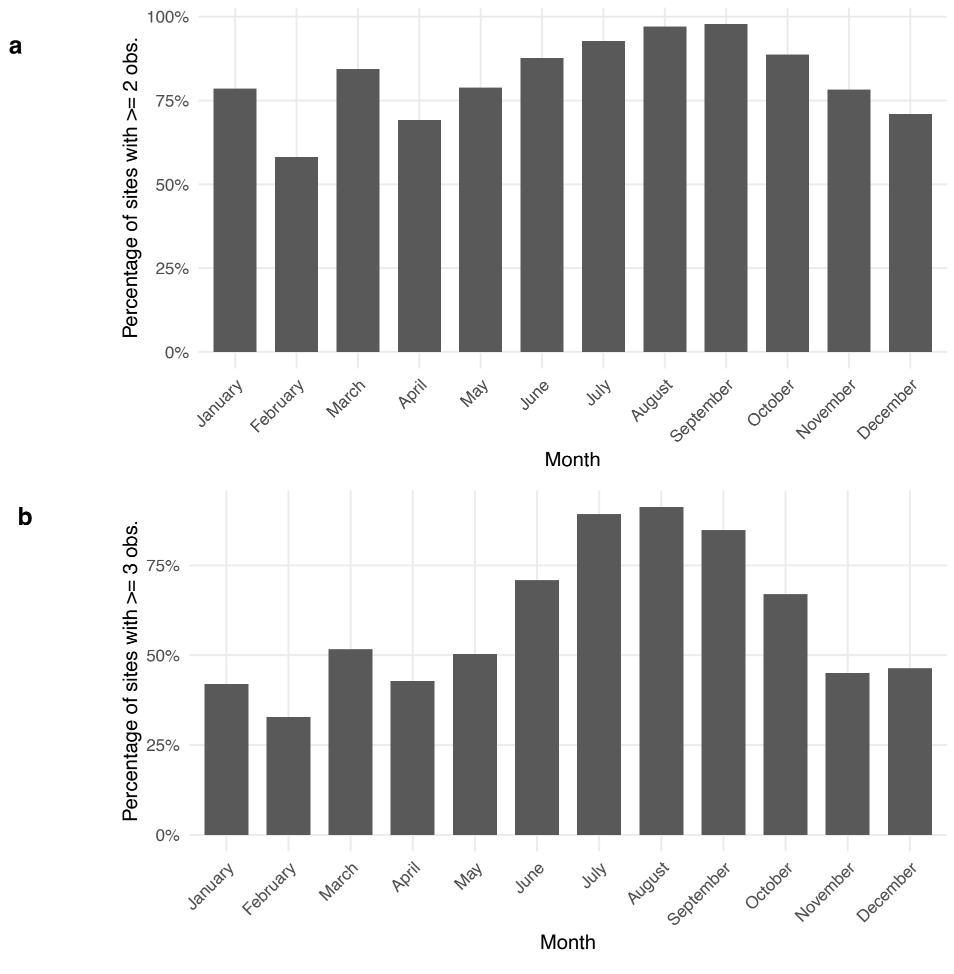

Figure S13 | Seasonal pattern of Sentinel-2 data availability across Amazon basin study plots in 2019. For each calendar month, the panels show the percentage of sites with **(a)** at least two and **(b)** at least three cloud-free

Sentinel-2 observations. These diagnostics quantify the fraction of plot-month combinations for which the coefficient of variation (CV) of spectral reflectance could be reliably estimated.

***1.3.2 Mixed-effects models including data-availability* -** To test whether uneven data availability biases the relationship between leaf traits and reflectance stability, we repeated our main mixed-effects model (M1) of dry-season CV of blue reflectance including the number of valid observations per site-month as an explicit covariate (M2). In M2, the effect of n_obs on CV of blue was positive but small (2.28 ± 0.95 % CV per additional observation), consistent with slightly higher estimated variability in site-months with more frequent cloud-free acquisitions. Crucially, the SLA-CV relationship remained positive and of similar magnitude (0.24 ± 0.11 in M1 vs 0.26 ± 0.11 in M2; Table S2). This indicates that uneven sampling and masking primarily add noise rather than spuriously strengthening the SLA-CV signal.

***1.3.3 High-coverage subset analysis* -** As an additional robustness check, we repeated the analysis using only "high-coverage" site-month combinations with n_obs ≥ 3. This high-coverage subset retains observations from all months and both seasons, and the seasonal distribution of valid acquisitions closely matches that of the full dataset. For this subset, the best-supported model (M3) included SLA and season as fixed effects. In this model, SLA again showed a positive association with CV of blue reflectance (0.20 ± 0.12), of comparable effect size to the full-dataset models (M1 and M2), though with slightly wider confidence intervals due to the reduced sample size (Table S2).

Taken together, these diagnostics show that: (i) a large majority of site-month combinations meet the minimum data-availability threshold for computing CV of blue reflectance; (ii) seasonal variation in cloud masking produces the expected pattern of higher coverage during the dry season but does not eliminate entire months or seasons from the analysis; (iii) explicitly accounting for data availability (n_obs) in the mixed-effects models, and restricting the analysis to high-coverage site-months, leaves the SLA-reflectance-stability relationship qualitatively unchanged. We therefore conclude that while data availability explains a small fraction of variance in CV, the main trait-reflectance relationships reported in the manuscript are robust to seasonal masking and uneven sampling.
